# Influence of hospital capabilities and prehospital time on outcomes of thrombectomy for stroke in Japan from 2013 to 2016

**DOI:** 10.1038/s41598-022-06074-1

**Published:** 2022-02-28

**Authors:** Ai Kurogi, Daisuke Onozuka, Akihito Hagihara, Kunihiro Nishimura, Akiko Kada, Manabu Hasegawa, Takahiro Higashi, Takanari Kitazono, Tsuyoshi Ohta, Nobuyuki Sakai, Hajime Arai, Susumu Miyamoto, Tetsuya Sakamoto, Koji Iihara, the J-ASPECT Study Collaborators, Masayoshi Takigami, Kenji Kamiyama, Kiyohiro Houkin, Shogo Nishi, Sadao Kaneko, Koji Oka, Yusuke Nakagaki, Hiroshi Ooyama, Katsumi Takizawa, Naoki Tokumitsu, Susumu Suzuki, Nozomi Suzuki, Teruo Kimura, Naoto Izumi, Kazumi Nitta, Masafumi Ohtaki, Masanori Isobe, Mikio Nishiya, Mitsunobu Kaijima, Syouji Mabuchi, Kuniaki Ogasawara, Naohiko Kubo, Yukihiko Shimizu, Keiichi Saito, Tatumi Yamanome, Akinori Yabuta, Atsuo Yoshino, Junichi Harashina, Masami Shimoda, Hiroyuki Jimbo, Hideki Murakami, Hiroyuki Masaoka, Hirotoshi Ohtaka, Hiroki Yoshida, Ichiro Suzuki, Michihiro Kohno, Yoshinori Arai, Akira Isoshima, Mitsuhiko Hokari, Kensuke Kawai, Taketoshi Maehara, Hajime Arai, Takakazu Kawamata, Makoto Noguchi, Haruhiko Hoshino, Hirofumi Hiyama, Kensaku Yoshida, Mitsuyuki Fujitsuka, Yasuaki Takeda, Hirohide Karasudani, Shiro Kobayashi, Michio Nakamura, Junichi Ono, Sumio Suda, Hiromu Hadeishi, Kenji Wakui, Hirokazu Tanno, Naoaki Sato, Hideki Sakai, Takashi Matsumoto, Naoki Koketsu, Ichiro Nakahara, Toshinori Hasegawa, Naoto Kuwayama, Nobuhiko Mizutani, Noriyuki Suzaki, Keizo Yasui, Akira Ikeda, Youtarou Takeuchi, Toshihiko Wakabayashi, Hisashi Tanaka, Junpei Yoshimoto, Ogura Koichiro, Toshio Yokoe, Kenichi Murao, Tomonori Yamada, Amami Kato, Akatsuki Wakayama, Hiroharu Kataoka, Kouich Iwatsuki, Yoshikazu Nakajima, Hidefuku Gi, Ryunosuke Uranishi, Yusaku Nakamura, Kazunori Yamanaka, Hiroyuki Matsumoto, Hiroaki Fujiwara, Yoshiyasu Iwai, Masashi Morikawa, Kazuyuki Tane, Kazuo Hashikawa, Shunichi Yoneda, Kohsuke Yamashita, Masahiko Kitano, Kazuhito Nakamura, Katsuhiko Kono, Kenji Ohata, Toshihiko Kuroiwa, Kazusige Maeno, Motohiro Arai, Masaaki Iwase, Kenji Hashimoto, Takashi Tsuruno, Shinichiro Kurokawa, Takeshi Matsuyama, Takamichi Yuguchi, Yoshihumi Teramoto, Takayuki Matsuo, Naoki Kitagawa, Makio Kaminogo, Seisaburo Sakamoto, Yoshiharu Tokunaga, Ei-Ichirou Urasaki, Junichi Kuratsu, Akira Takada, Shu Hasegawa, Toru Nishi, Isao Fuwa, Hiromasa Tsuiki, Hiromasa Tsuiki, Kazunari Koga, Hiroshi Egami, Tadao Kawamura, Makoto Goda, Yu Takeda, Yasuyuki Nagai, Masaki Morisige, Yutaka Yamaguchi, Shiro Miyata, Hideo Takeshima, Kazutaka Yatsushiro, Hajime Ohta, Kazuho Hirahara, Teruaki Kawano, Souichi Obara, Hiroshi Seto, Shunichi Tanaka, Koiti Moroki, Kazunori Arita, Shogo Ishiuchi, Toshimitsu Uchihara, Susumu Mekaru, Tomoaki Nagamine, Jin Momoji, Satoshi Yamamoto, Atusi Kimoto, Tsutomu Kadekaru, Akihiko Saito, Osamu Onodera, Hideaki Takahashi, Hiroyuki Arai, Shigekazu Takeuchi, Hiroki Takano, Osamu Fukuda, Mitsuo Kouno, Igarashi Michitoku, Michiya Kubo, Hiroaki Hondo, Miyamori Tadao, Ryouichi Masuda, Takata Hisashi, Toru Masuoka, Naoki Shirasaki, Hisashi Nitta, Makoto Kimura, Hisato Minamide, Shunsuke Shiraga, Mitsutoshi Nakada, Shuji Sato, Hiroki Toda, Osamu Yamamura, Masanori Kabuto, Jyunya Hayashi, Hiroyuki Kinouchi, Toyoaki Shinohara, Hidehito Koizumi, Mikito Uchida, Syougo Imae, Hiroshi Ozawa, Osamu Nishizaki, Masakazu Suga, Kanehisa Kohno, Kiichiro Zenke, Hiromichi Sadashima, Hikaru Mizobuchi, Satoru Hayashi, Masanori Morimoto, Takeshi Kohno, Tetsuya Ueba, Hiroyuki Nishimura, Norihito Shirakawa, Masahiro Kagawa, Naoki Hayashi, Atsushi Shindo, Kimihiro Yoshino, Tetsuya Masaoka, Kenwakai Otemati Hospital, Ichiro Nakahara, Akira Nakamizo, Yuji Okamoto, Shigenari Kin, Haruki Takahashi, Satoshi Suzuki, Koji Iihara, Katsuyuki Hirakawa, Akira Nakamizo, Akio Ookura, Koichirou Matsukado, Hidenori Yoshida, Hiroshi Nakane, Isao Inoue, Kei Hisada, Tsutomu Hitotsumatsu, Kouichi Kuramoto, Junya Hayashi, Hiromichi Ooishi, Masani Nonaka, Motohiro Morioka, Haruhisa Tsukamoto, Hiroshi Sugimori, Shinichirou Ishihara, Nobuaki Momozaki, Masayuki Miyazono, Akihiro Nemoto, Nobuo Hirota, Hiroaki Tanaka, Hiroshi Tanaka, Atsushi Tsuchiya, Katsumi Sakata, Hidetoshi Murata, Motohiro Nomura, Hitoshi Ozawa, Kotaro Tsumura, Makoto Inaba, Taturou Mori, Tomoaki Terada, Takahisa Mori, Masato Sugitani, Yuichiro Tanaka, Masaru Yamada, Mitsunori Matsumae, Keiichirou Onitsuka, Kosuke Miyahara, Sumio Endou, Atsuhiro Kojima, Shinichi Yagi, Hidekazu Takahashi, Hiroyuki Kaidu, Akira Tsunoda, Kyoichi Nomura, Takamitsu Fujimaki, Hidetoshi Ooigawa, Masahiko Tanaka, Hiroshi Wanihuchi, Hirochiyo Wada, Akio Hyodo, Ken Asakura, Akazi Kazunori, Hideyuki Kurihara, Shigehiro Ohmori, Hiroshi Kusunoki, Satoshi Magarisawa, Shinichi Okabe, Shinji Yamamoto, Hiroko Oyama, Shin Tsuruoka, Mikihiko Takeshita, Akira Matsumura, Kazuya Uemura, Hitoshi Tabata, Keishi Fujita, Masashi Nakatsukasa, Norifumi Shimoeda, Hideo Kunimine, Masayuki Ishihara, Kazuhiro Kikuchi, Nozomu Murai, Warou Taki, Nobukuni Murakami, Minoru Kidooka, Yoshihiro Iwamoto, Hiroshi Tenjin, Kouji Shiga, Nobuhito Mori, Eiji Kohmura, Takeshi Kondoh, Haruo Yamashita, Keigo Matsumoto, Naoya Takeda, Takayuki Sakaki, Hiroji Miyake, Eiichiro Mabuchi, Masayuki Yokota, No, Hideyuki Ohnishi, Masaaki Saiki, Minoru Asahi, Junji Koyama, Yoshio Sakagami, Shinya Noda, Junichi Iida, Tetsuya Morimoto, Hiroyuki Nakase, Hidehiro Hirabayashi, Naoyuki Nakao, Toshikazu Kuwata, Yoshinari Nakamura, Hiroshi Ishiguchi, Teruyuki Habu, Masamichi Kurosaki, Hiroki Ohkuma, Seiko Hasegawa, Hiromu Konno, Atsuhito Takemura, Atsuya Okubo, Hitoshi Saito, Junta Moroi, Hiroaki Shimizu, Masayuki Sasou, Yoichi Watanabe, Kiyoshi Saito, Masahiro Satoh, Zenichiro Watanabe, Takayuki Koizumi, Shoji Mashiyama, Tomoyoshi Oikawa, Sonoda Yukihiko, Rei Kondo, Atsuo Shinoda, Eiichiro Kamatsuka, Keiten So, Toshihiko Kinjo, Kennji Itou, Yohei Kudoh, Kazuhiko Sato, Arai Hiroaki, Hidenori Endo, Hiroshi Karibe, Kou Takahashi, Masayuki Nakajima, Kazuyoshi Watanabe, Kazuhiko Nozaki, Motohiro Takayama, Tarou Komuro, Fumio Suzuki, Hidenori Suzuki, Hiroto Murata, Fumitaka Miya, Seiji Fukazawa, Seiya Takehara, Yoshihiko Watanabe, Teiji Nakayama, Haruhiko Sato, Shinji Amano, Katsuhiro Kuroda, Akira Morooka, Takafumi Wataya, Tetsuya Tanigawara, Toru Iwama, Junki Ito, Shinji Noda, Kazuyuki Kouno, Kazuo Kitazawa, Yoshikazu Kusano, Masanobu Hokama, Hiroki Sato, Sumio Kobayashi, Shinsuke Muraoka, Masaki Miyatake, Kensuke Hayashida, Keiichi Sakai, Fusao Ikawa, Gen Ishida, Takato Kagawa, Youichirou Namba, Hiroyuki Nakashima, Koji Tokunaga, Isao Date, Masaaki Uno, Masaki Chin, Hidemichi Sasayama, Hideyuki Yoshida, Akira Watanabe, Kunihiko Harada, Manabu Urakawa, Yasuhiro Hamada, Michiyasu Suzuki, Takafumi Nishizaki, Katsuhiro Yamashita, Ryuji Nakamura, Masayuki Sumida, Shinichi Wakabayashi, Kaoru Kurisu, Atsushi Tominaga, Masaaki Shibukawa, Kawamoto Yukihiko, Shinji Okita, Kenjirou Hujiwara, Takashi Matsuoka, Osamu Hamasaki, Junichiro Satomi, Masahito Agawa, Hirofumi Oka, Kunikazu Yoshimura, Sei Haga, Katsuyuki Asaoka, Toshitaka Nakamura, Makoto Takeda, Nobuaki Kobayasi, Satoshi Ushikoshi, Nobuhiro Mikuni, Jun Niwa, Rokuya Tanikawa, Akinori Yamamura, Akira Takahashi, Noriaki Watabe, Junkoh Sasaki, Yasunari Otawara, Kazuyuki Miura, Teiji Tominaga, Tatsuya Sasaki, Takayuki Sugawara, Masayuki Ezura, Kenji Yamamoto, Syuichi Ishikawa, Yoshida Masahiro, Sunao Takemura, Masahisa Kawakami, Satoshi Ihara, Yasushi Shibata, Takashi Saegusa, Toshihiko Iuchi, Chiaki Ito, Seiichiro Hoshi, Sumio Isimaru, Osamu Okuda, Kazunari Yoshida, Takekazu Akiyama, Masateru Katayama, Masahiko Kasai, Tomonori Kobayashi, Oikawa Akihiro, Naohisa Miura, Osamu Tao, Takahiro Oota, Atumi Takenobu, Toshihiro Kumabe, Sachio Suzuki, Takashi Kumagai, Keiichi Nishimaki, Kazuhiro Hongo, Yasuyuki Toba, Kuroyanagi Takayuki, Hiroaki Shigeta, Atsushi Sato, Satoshi Kuroda, Cheho Park, Sotaro Higashi, Hirofumi Oyama, Kazuyoshi Hattori, Yoichi Uozumi, Norimoto Nakahara, Mitsuhito Mase, Nobukazu Hashimoto, Toshikazu Ichihashi, Katsunobu Takenaka, Shinichi Shirakami, Yoshinari Okumura, Kazuhiro Yokoyama, Susumu Miyamoto, Yoshinori Akiyama, Kenji Hashimoto, Masaaki Saiki, Kazuo Yamamoto, Naofumi Isono, Tsugumichi Ichioka, Nakazawa Kazutomo, Misao Nishikawa, Tsuyoshi Inoue, Manabu Kinoshita, Shinichi Yoshimura, Minoru Saitoh, Hideo Aihara, Hajimu Miyake, Kazuyuki Kuwayama, Kotaro Ogihara, Shigeki Nishino, Yasuyuki Miyoshi, Tadashi Arisawa, Shigeru Daido, Kimihisa Kinoshita, Keisuke Migita, Keiichi Akatsuka, Hirosuke Fujisawa, Junkoh Yamamoto, Yosimasa Kinosita, Satoshi Inoha, Hitonori Takaba, Tadahisa Shono, Hitoshi Tsugu, Shuji Hayashi, Tatsuya Abe, Susumu Nakashima, Takehisa Tuji, Keizou Yamamoto, Akihiko Kaga, Reizou Kanemaru, Koji Takasaki, Junichi Imamura, Masahiro Noha, Saburo Watanabe, Nobuyuki Sakai, Hiroaki Minami, Tomoyoshi Okumura, Shinjitsu Nishimura, Shinichi Numazawa, Yasunari Niimi, Isao Kitahara, Hidemitu Nakagawa, Kiyoshi Kazekawa, Kouzou Fukuyama, Makoto Ichinose, Koji Matuoka, Yasuhiro Fujimoto, Youichi Hashimoto, Takeshi Matsuoka, Takamitsu Uchizawa, Tomohiko Satou, Makoto Hasebe, Tomoaki Kameda, Hiroaki Sawaura, Takayuki Kubodera, Satoshi Utsuki, Kazuaki Awamori, Chiaki Takahashi, Kazumasa Yamatani, Toshiyuki Tsukada, Ryoichi Hayashi, Masakazu Kitahara, Yukinari Kakizawa, Yasumasa Yamamoto, Takashi Yoshida, Shinji Okumura, Yasunobu Gotou, Takashi Tominaga, Hirotoshi Hamaguchi, Nozomi Mori, Naoki Shinohara, Yasushi Ejima, Mayumi Mori, Hitoshi Miyake, Masaru Idei, Yoshihiro Nishiura, Hiromichi Koga, Kazuya Morimoto, Jae-Hyun Son, Yoshimasa Niiya, Tsuneo Shishido, Mamoru Murakami, Takaaki Yoshida, Masahito Hara, Tatsuya Nakamura, Takuya Kawai, Takashi Inoue, Isao Sasaki, Katsuhiko Hayashi, Ichiro Fujishima, Naoko Fujimura, Seiko Kataoka, Masayuki Yokota

**Affiliations:** 1 Department of Neurosurgery, Graduate School of Medical Sciences, Kyushu University, Fukuoka, Japan; 2 Department of Preventive Medicine and Epidemiology, National Cerebral and Cardiovascular Center, Suita, Japan; 3 Department of Clinical Research Planning and Management, Clinical Research Center, National Hospital Organization Nagoya Medical Centre, Nagoya, Japan; 4 Immunization Office, Health Service Division, Health Service Bureau, Ministry of Health, Labor and Welfare, Tokyo, Japan; 5 Division of Health Services Research, Center for Cancer Control and Information Services, National Cancer Center, Tokyo, Japan; 6 Department of Medicine and Clinical Science, Graduate School of Medical Science, Kyushu University, Fukuoka, Japan; 7 Department of Neurosurgery, National Cerebral and Cardiovascular Center, Suita, Japan; 8 Department of Neurosurgery, Kobe City Medical Centre General Hospital, Kobe, Japan; 9 Department of Neurosurgery, Juntendo University School of Medicine, Tokyo, Japan; 10 Department of Neurosurgery, Kyoto University Graduate School of Medicine, Kyoto, Japan; 11 Department of Emergency Medicine, Teikyo University School of Medicine, Tokyo, Japan; 12 Department of Neurosurgery, Director General of the Hospital, National Cerebral and Cardiovascular Center, 6-1, Kishibe-shimmmachi, Suita, Osaka 564-8565 Japan; 13 Sapporo City General Hospital, Sapporo, Japan; 14 Nakamura Memorial Hospital, Sapporo, Japan; 15 Hokkaido University Hospital, Sapporo, Japan; 16 Sapporo Higashi Tokushukai Hospital, Sapporo, Japan; 17 Kashiwaba Neurosurgical Hospital, Sapporo, Japan; 18 Nakamura Memorial South Hospital, Sapporo, Japan; 19 Takikawa Neurosurgical Hospital, Takikawa, Japan; 20 Muroran City General Hospital, Muroran, Japan; 21 Japanese Red Cross Asahikawa Hospital, Asahikawa, Japan; 22 Nayoro City Hospital, Nayoro, Japan; 23 Rumoi Central Clinic, Rumoi, Japan; 24 Japanese Red Cross Kitami Hospital, Kitami, Japan; 25 Doutou Neurosurgical Hospital, Kitami, Japan; 26 Meiseikai Abashiri Neurosurgical Rehabilitation Hospital, Abashiri, Japan; 27 Hokuto Hospital, Obihiro, Japan; 28 Obihiro Kosei General Hospital, Obihiro, Japan; 29 Kushiro Rosai Hospital, Kushiro, Japan; 30 Hakodate Neurosurgical Hospital, Hakodate, Japan; 31 Hokushikai Megumino Hospital, Eniwa, Japan; 32 Otaru Municipal Medical Center for Brain Cardiovascular and Mental Disorders, Otaru, Japan; 33 Iwate Medical University Hospital, Shiwa, Japan; 34 Morioka Red Cross Hospital, Morioka, Japan; 35 Iwate Prefectural Isawa Hospital, Oshu, Japan; 36 Iwate Prefectural Iwai Hospital, Ichinoseki, Japan; 37 Iwate Prefectural Oofunato Hospital, Ohunato, Japan; 38 Iwate Prefectural Ninohe Hospital, Ninohe, Japan; 39 Nihon University Itabashi Hospital, Tokyo, Japan; 40 Juntendo University Nerima Hospital, Tokyo, Japan; 41 Kasai Shoikai Hospital, Tokyo, Japan; 42 Tokai University Hachioji Hospital, Hachioji, Japan; 43 Tokyo Medical University Hachioji Medical Center, Hachioji, Japan; 44 Inagi Municipal Hospital, Inagi, Japan; 45 NHO Disaster Medical Center, Tachikawa, Japan; 46 Higashiyamato Hospital, Higashiyamato, Japan; 47 Kyorin University Hospital, Mitaka, Japan; 48 Japanese Red Cross Medical Center, Tokyo, Japan; 49 Tokyo Medical University Hospital, Tokyo, Japan; 50 Makita General Hospital, Tokyo, Japan; 51 Omori Red Cross Hospital, Tokyo, Japan; 52 Nitobe Memorial Nakano General Hospital, Tokyo, Japan; 53 NTT Medical Center Tokyo, Tokyo, Japan; 54 Tokyo Medical and Dental University Hospital, Tokyo, Japan; 55 Juntendo University Hospital, Tokyo, Japan; 56 Tokyo Womens Medical University Hosptal, Tokyo, Japan; 57 Tokyo Teisin Hospital, Tokyo, Japan; 58 Tokyo Saiseikai Central Hospital, Tokyo, Japan; 59 JCHO Tokyo Takanawa Hospital, Tokyo, Japan; 60 Tokyo Metropolitan Hiroo Hospital, Tokyo, Japan; 61 Kugayama Hospital, Tokyo, Japan; 62 JCHO Tokyo Yamate Medical Center, Tokyo, Japan; 63 Matsudo City Hospital, Matsudo, Japan; 64 Nippon Medical School Chiba Hokusoh Hospital, Inzai, Japan; 65 Narita Red Cross Hospital, Narita, Japan; 66 Chiba Cerebral and Cardiovascular Center, Ichihawa, Japan; 67 Kimitsu Chuo Hospital, Kisarazu, Japan; 68 Kameda Medical Center, Kamogawa, Japan; 69 Chiba Neurosurgical Clinic, Chiba, Japan; 70 NHO Chiba Medical Center, Chiba, Japan; 71 Kobari General Hospital, Noda, Japan; 72 NHO Toyohashi Medical Center, Toyohashi, Japan; 73 Toyokawa City Hospital, Toyokawa, Japan; 74 Tosei General Hospital, Seto, Japan; 75 Fujita Health University Hospital, Toyoake, Japan; 76 Komaki City Hospital, Komaki, Japan; 77 Kasugai Municipal Hospital, Kasugai, Japan; 78 Konan Kosei Hospital, Konan, Japan; 79 NHO Nagoya Medical Center, Nagoya, Japan; 80 Nagoya Daini Red Cross Hospital, Nagoya, Japan; 81 JCHO Chukyo Hospital, Nagoya, Japan; 82 Meitetsu Hospital, Nagoya, Japan; 83 Nagoya University Hospital, Nagoya, Japan; 84 Minato Medical Coop-Kyoritsu General Hospital, Nagoya, Japan; 85 Nagoya Memorial Hospital, Nagoya, Japan; 86 Toyota Kosei Hospital, Toyota, Japan; 87 Kainan Hospital, Yatomi, Japan; 88 Nishio Municipal Hospital, Nishio, Japan; 89 Hekinan Municipal Hospital, Hekinan, Japan; 90 Shiroyama Hospital, Habikino, Japan; 91 NHO Osakaminami Medical Center, Kawachinagano, Japan; 92 Kindai University Hospital, Osakasayama, Japan; 93 Osaka Neurological Institute, Toyonaka, Japan; 94 National Cerebral and Cardiovascular Center, Suita, Japan; 95 Osaka University Hospital, Suita, Japan; 96 Sakai City Medical Center, Sakai, Japan; 97 Baba Memorial Hospital, Sakai, Japan; 98 Bellland General Hospital, Sakai, Japan; 99 Kindai University Sakai Hospital, Sakai, Japan; 100 Fuchu Hospital, Izumi, Japan; 101 Kishiwada Tokushukai Hospital, Kishiwada, Japan; 102 Meisei Hospital, Osaka, Japan; 103 Osaka City General Hospital, Osaka, Japan; 104 Yodogawa Christian Hospital, Osaka, Japan; 105 Osaka Kouseinenkin Hospital, Osaka, Japan; 106 Tane General Hospital, Osaka, Japan; 107 NHO Osaka National Hospital, Osaka, Japan; 108 Nipponbashi Hospital, Osaka, Japan; 109 Osaka Red Cross Hospital, Osaka, Japan; 110 Tominaga Hospital, Osaka, Japan; 111 Suisyoukai Murata Hospital, Osaka, Japan; 112 Saiseikai Noe Hospital, Osaka, Japan; 113 Osaka City University Hospital, Osaka, Japan; 114 Osaka Medical College Hospital, Takatsuki, Japan; 115 Takatsuki General Hospital, Takatsuki, Japan; 116 Midorigaoka Hospital, Takatsuki, Japan; 117 Kansai Medical University Takii Hospital, Moriguchi, Japan; 118 Matsushita Memorial Hospital, Moriguchi, Japan; 119 Yao Tokushukai General Hospital, Yao, Japan; 120 Ishinkai Yao General Hospital, Yao, Japan; 121 Wakakusa Daiichi Hospital, Higashiosaka, Japan; 122 Hanwa Memorial Hospital, Osaka, Japan; 123 Yuaikai Hospital, Osaka, Japan; 124 Nagasaki University Hospital, Nagasaki, Japan; 125 Juzenkai Hospital, Nagasaki, Japan; 126 Saiseikai Nagasaki Hospital, Nagasaki, Japan; 127 Sasebo City General Hospital, Sasebo, Japan; 128 Sasebo Chuo Hospital, Sasebo, Japan; 129 Nagasakiken Shimabara Hospital, Shimabara, Japan; 130 NHO Nagasaki Kawatana Medical Center, Kawatana, Japan; 131 Kumamoto University Hospital, Kumamoto, Japan; 132 Kumamoto City Hospital, Kumamoto, Japan; 133 Japanese Red Cross Kumamoto Hospital, Kumamoto, Japan; 134 Saiseikai Kumamoto Hospital, Kumamoto, Japan; 135 Arao Municipal Hospital, Arao, Japan; 136 Kumamoto Rousai Hospital, Yatsushiro, Japan; 137 Minamata City General Hospital and Medical Center, Minamata, Japan; 138 JCHO Kumamoto General Hospital, Yatsushiro, Japan; 139 Uki General Hospital, Uki, Japan; 140 Tenshindo Hetsugi Hospital, Oita, Japan; 141 Almeida Memorial Hospital, Oita, Japan; 142 Oita Prefectural Hospital, Oita, Japan; 143 NHO Beppu Medical Center, Beppu, Japan; 144 JCHO Nankai Medical Center, Saiki, Japan; 145 Oitaoka Hospital, Oita, Japan; 146 Seiwakai Wada Hospital, Hyuga, Japan; 147 University of Miyazaki Hospital, Miyazaki, Japan; 148 Fujimoto General Hospital, Miyakonojo, Japan; 149 Miyakonojo Medical Association Hospital, Miyakonojo, Japan; 150 Kagoshima City Hospital, Kagoshima, Japan; 151 Kagoshima Tokushukai Hospital, Kagoshima, Japan; 152 Obara Hospital, Makurazaki, Japan; 153 Izumi General Medical Center, Izumi, Japan; 154 Kagoshima Prefectural Kanoya Medical Center, Kanoya, Japan; 155 Tokuda Neurosurgical Hospital, Kanoya, Japan; 156 Kagoshima University Hospital, Kagoshima, Japan; 157 University of the Ryukyus Hospital, Nakagami, Japan; 158 Okinawa Prefectural Hokubu Hospital, Nago, Japan; 159 Urasoe General Hospital, Urasoe, Japan; 160 Okinawa Prefectural Nanbu Medical Center/Nanbu Child Medical Center, Shimajiri, Japan; 161 Naha City Hospital, Naha, Japan; 162 Okinawa Miyako Hospital, Miyakojima, Japan; 163 Okinawa Kyodo Hospital, Naha, Japan; 164 Nanbu Tokushukai Hospital, Shimajiri, Japan; 165 Niigata City General Hospital, Niigata, Japan; 166 Niigata University Medical & Dental Hospital, Niigata, Japan; 167 Niigata Cancer Center Hospital, Niigata, Japan; 168 Niigata Neurosurgical Hospital, Niigata, Japan; 169 Nagaoka Chuo General Hospital, Nagaoka, Japan; 170 Tachikawa General Hospital, Nagaoka, Japan; 171 Saito Memorial Hospital, Minamiuonuma, Japan; 172 Niigata Tokamachi Hospital, Tokamachi, Japan; 173 Niigata Prefectural Central Hospital, Joetsu, Japan; 174 Saiseikai Toyama Hospital, Toyama, Japan; 175 Toyama Prefectural Central Hospital, Toyama, Japan; 176 Toyama City Hospital, Toyama, Japan; 177 Yatsuo General Hospital, Toyama, Japan; 178 Himi Municipal Hospital, Himi, Japan; 179 Tonami General Hospital, Tonami, Japan; 180 Shinseikai Toyama Hospital, Imizu, Japan; 181 Kaga Medical Center, Kaga, Japan; 182 Komatsu Municipal Hospital, Komatsu, Japan; 183 Yawata Medical Center, Komatsu, Japan; 184 Ishikawa Prefectural Central Hospital, Kanazawa, Japan; 185 Kanazawa Medical University Hospital, Kahoku, Japan; 186 Kanazawa University Hospital, Kanazawa, Japan; 187 Kanazawa Neurosurgical Hospital, Nonoichi, Japan; 188 Japanese Red Cross Fukui Hospital, Fukui, Japan; 189 University of Fukui Hospital, Yoshida, Japan; 190 Fukui Katsuyama General Hospital, Katsuyama, Japan; 191 Hayashi Hospital, Echizen, Japan; 192 Tannan Regional Medical Center, Sabae, Japan; 193 University of Yamanashi Hospital, Chuo, Japan; 194 Kofu Neurosurgical Hospital, Kofu, Japan; 195 Yamanashi Prefectural Central Hospital, Kofu, Japan; 196 Yamanashi Kosei Hospital, Yamanashi, Japan; 197 Fujiyoshida Municipal Hospital, Fujiyoshida, Japan; 198 Yamanashi Red Cross Hospital, Minamitsuru, Japan; 199 Saiseikai Imabari Hospital, Imabari, Japan; 200 Matsuyama Shimin Hospital, Matsuyama, Japan; 201 Ehime Prefectural Central Hospital, Matsuyama, Japan; 202 Uwajima City Hospital, Uwajima, Japan; 203 Okinawatokushuukai Uwajimatokushukai Hospital, Uwajima, Japan; 204 Izumino Hospital, Kochi, Japan; 205 Chikamori Hospital, Kochi, Japan; 206 Kochi Health Sciences Center, Kochi, Japan; 207 Japanese Red Cross Kochi Hospital, Kochi, Japan; 208 Kochi Medical School Hospital, Nankoku, Japan; 209 Hata Kenmin Hospital, Sukumo, Japan; 210 Usuikai Tano Hospital, Aki, Japan; 211 Takamatsu Municipal Hospital, Takamatsu, Japan; 212 Takamatsu Red Cross Hospital, Takamatsu, Japan; 213 Osaka Neurosurgical Hospital, Takamatsu, Japan; 214 Kagawa University Hospital, Kita, Japan; 215 Kagawa Rosai Hospital, Marugame, Japan; 216 Mitoyo General Hospital, Kanonji, Japan; 217 Kenwakai Otemati Hospital, Kitakyushu, Japan; 218 Kokura Memorial Hospital, Kitakyushu, Japan; 219 Steel Memorial Yawata Hospital, Kitakyushu, Japan; 220 Saiseikai Yahata General Hospital, Kitakyushu, Japan; 221 Fukuokashinmizumaki Hospiral, Onga, Japan; 222 Obase Hospital, Miyako, Japan; 223 Kieikai Hospital, Fukuoka, Japan; 224 Kyushu University Hospital, Fukuoka, Japan; 225 Fukuoka City Hospital, Fukuoka, Japan; 226 NHO Kyushu Medical Center, Fukuoka, Japan; 227 Saiseikai Fukuoka General Hospital, Fukuoka, Japan; 228 Hamanomachi Hospital, Fukuoka, Japan; 229 Fukuoka Tokushukai Medical Center, Kasuga, Japan; 230 NHO Fukuoka Higashi Medical Center, Koga, Japan; 231 Fukuoka Seisyukai Hospital, Kasuya, Japan; 232 Hachisuga Hospital, Munakata, Japan; 233 Shin Koga Hospital, Kurume, Japan; 234 Omuta City Hospital, Omuta, Japan; 235 Kawasaki Hospital, Yame, Japan; 236 Tanushimaru Central Hospital, Kurume, Japan; 237 Fukuoka University Hospital, Fukuoka, Japan; 238 Kurume University Hospital, Kurume, Japan; 239 Kitakyushu Municipal Medical Center, Kitakyushu, Japan; 240 Saga-Ken Medical Centre Koseikan, Saga, Japan; 241 Yayoigaoka Kage Hospital, Tosu, Japan; 242 Imari Arita Kyoritsu Hospital, Nishimatsuura, Japan; 243 NHO Ureshino Medical Center, Ureshino, Japan; 244 Yokohamashintoshi Neurosurgecal Hospital, Yokohama, Japan; 245 Yokosuka City Uwamachi Hospital, Yokosuka, Japan; 246 Yokohama City Minato Red Cross Hospital, Yokohama, Japan; 247 Yokohama Rosai Hospital, Yokohama, Japan; 248 Chigasaki Municipal Hospital, Chigasaki, Japan; 249 Kanto Rosai Hospital, Kawasaki, Japan; 250 Yokohama City University Medical Center, Yokohama, Japan; 251 Yokohama City University Hospital, Yokohama, Japan; 252 Yokohama Sakae Kyosai Hospital, Yokohama, Japan; 253 Ushioda General Hospital, Yokohama, Japan; 254 Sekishinkai Kawasakisaiwai Hospital, Kawasaki, Japan; 255 Saiseikai Yokohamashi Tobu Hospital, Yokohama, Japan; 256 JCHO Yokohama Chuo Hospital, Yokohama, Japan; 257 Showa University Fujigaoka Hospital, Yokohama, Japan; 258 Shonan Kamakura General Hospital, Kamakura, Japan; 259 Sagamihara Kyodo Hospital, Sagamihara, Japan; 260 St.Marianna University School of Medicine, Kawasaki, Japan; 261 Yamato Municipal Hospital, Yamato, Japan; 262 Tokai University Hospital, Isehara, Japan; 263 Tomei Atsugi Hospital, Atsugi, Japan; 264 NHO Yokohama Medical Center, Yokohama, Japan; 265 Yokohamasinmidori Hospital, Yokohama, Japan; 266 Saitama City Hospital, Saitama, Japan; 267 Kanto Neurosurgical Hospital, Kumagaya, Japan; 268 Ageo Central General Hospital, Ageo, Japan; 269 Musashino General Hospital, Kawagoe, Japan; 270 Koshigaya Municipal Hospital, Koshigaya, Japan; 271 Saitama Medical Center, Kawagoe, Japan; 272 Saitama Medical University Hospital, Iruma, Japan; 273 Saitama Cardiovascular and Respiratory Center, Kumagaya, Japan; 274 Kan-Etsu Hospital, Tsurugashima, Japan; 275 Saiseikai Kurihashi Hospital, Kuki, Japan; 276 Fukaya Red Cross Hospital, Fukaya, Japan; 277 Dokkyo Medical University Koshigaya Hospital, Koshigaya, Japan; 278 Japanese Red Cross Maebashi Hospital, Maebashi, Japan; 279 Institute of Brain and Blood Vessels Mihara Memorial Hospital, Isesaki, Japan; 280 NHO Takasaki General Medical Center, Takasaki, Japan; 281 Kurosawa Hospital, Takasaki, Japan; 282 Nishiagatsuma Welfare Hospital, Agatsuma, Japan; 283 Kiryu Kosei General Hospital, Kiryu, Japan; 284 Ota Memorial Hospital, Ota, Japan; 285 Seirei Memorial Hospital, Hitachi, Japan; 286 Tsuchiura Kyodo General Hospital, Tsuchiura, Japan; 287 Ibaraki Prefectural Central Hospital, Kasama, Japan; 288 JA Toride Medical Center, Toride, Japan; 289 Ushiku Aiwa General Hospital, Ushiku, Japan; 290 University of Tsukuba Hospital, Tsukuba, Japan; 291 Tsukuba Medical Center Hospital, Tsukuba, Japan; 292 Tsuchiura Kyodo Hospital Namegata District Medical Center, Namegata, Japan; 293 Ibaraki Seinan Medical Center Hospital, Sashima, Japan; 294 Ashikaga Red Cross Hospital, Ashikaga, Japan; 295 Saiseikai Ustunomiya Hospital, Utsunomiya, Japan; 296 Shimotsuga General Hospital, Tochigi, Japan; 297 Fujii Neurosurgical Hospital, Utsunomiya, Japan; 298 NHO Tochigi Medical Center, Utsunomiya, Japan; 299 Kurosu Hospital, Sakura, Japan; 300 Kyoto City Hospital, Kyoto, Japan; 301 Takeda Hospital, Kyoto, Japan; 302 Saiseikai Kyoto Hospital, Nagaokakyo, Japan; 303 Kyoto Okamoto Memorial Hospital, Kuse, Japan; 304 Kyoto Yamashiro General Medical Center, Kizugawa, Japan; 305 Kyoto Second Red Cross Hospital, Kyoto, Japan; 306 Ayabe City Hospital, Ayabe, Japan; 307 Kyoto Min-Iren Chuo Hospital, Kyoto, Japan; 308 Kobe University Hospital, Kobe, Japan; 309 Shinsuma General Hospital, Kobe, Japan; 310 Kobe Red Cross Hospital, Kobe, Japan; 311 JCHO Kobe Central Hospital, Kobe, Japan; 312 Nishikobe Medical Center, Kobe, Japan; 313 Hyogo Prefectural Nishinomiya Hospital, Nishinomiya, Japan; 314 Nishinomiya Kyoritsu Neurosurgical Hospital, Nishinomiya, Japan; 315 Takarazuka City Hospital, Takarazuka, Japan; 316 The Veritas Hospital, Kawanishi, Japan; 317 Takarazuka Daiichi Hospital, Takarazuka, Japan; 318 Ohnishi Neurological Center, Akashi, Japan; 319 Nishiwaki Municipal Hospital, Nishiwaki, Japan; 320 Tsukazaki Hospital, Himeji, Japan; 321 NHO Himeji Medical Center, Himeji, Japan; 322 Ako City Hospital, Ako, Japan; 323 Toyooka Hospital, Toyooka, Japan; 324 Hyogo Prefectural Awaji Medical Center, Sumoto, Japan; 325 Itami Kousei Neurosurgical Hospital, Itami, Japan; 326 Nara Prefectural Nara Hospital, Nara, Japan; 327 Kouseikai Takai Hospital, Tenri, Japan; 328 Nara Medical University Hospital, Kashihara, Japan; 329 Saiseikai Gose Hospital, Gose, Japan; 330 NHO Nara Medical Center, Nara, Japan; 331 Wakayama Medical University Hospital, Wakayama, Japan; 332 Wakayama Rosai Hospital, Wakayama, Japan; 333 NHO Minami Wakayama Medical Center, Tanabe, Japan; 334 Shingu Municipal Medical Center, Shingu, Japan; 335 Wakayama-Seikyo Hospital, Wakayama, Japan; 336 Tottori University Hospital, Yonego, Japan; 337 Hirosaki University Hospital, Hirosaki, Japan; 338 Kurosishi General Hospital, Kuroishi, Japan; 339 Japanese Red Cross Society Hachinohe Hospital, Hachinohe, Japan; 340 Aomori City Hospital, Aomori, Japan; 341 Odate Municipal General Hospital, Odate, Japan; 342 Akita City Hospital, Akita, Japan; 343 Research Institute for Brain and Blood Vessels-Akita, Akita, Japan; 344 Akita University Hospital, Akita, Japan; 345 Kazuno Kosei Hospital, Kazuno, Japan; 346 Fukushima Red Cross Hospital, Fukuchima, Japan; 347 Fukushima Medical University Hospital, Fukuchima, Japan; 348 Fujita General Hospital, Date, Japan; 349 Southern Tohoku Hospital, Koriyama, Japan; 350 Takeda General Hospital, Aizuwakamatsu, Japan; 351 Iwaki Kyoritsu General Hospital, Iwaki, Japan; 352 Minamisoma City General Hospital, Minamisoma, Japan; 353 Yamagata University Hospital, Yamagata, Japan; 354 Yamagata City Hospital Saiseikan, Yamagata, Japan; 355 Shinoda General Hospital, Yamagata, Japan; 356 Kitamurayama Hospital, Higashine, Japan; 357 Yamagata Prefectural Shinjo Hospital, Shinjo, Japan; 358 Okitama Public General Hospital, Higashiokitama, Japan; 359 Yonezawa City Hospital, Yonezawa, Japan; 360 Sanyudo Hospital, Yonezawa, Japan; 361 Tsuruoka Municipal Shonai Hospital, Tsuruoka, Japan; 362 South Miyagi Medical Center, Shibata, Japan; 363 Kohnan Hospital, Sendai, Japan; 364 Sendai City Hospital, Sendai, Japan; 365 Furukawaseiryou Hospital, Osaki, Japan; 366 Omihachiman Community Medical Center, Omihachiman, Japan; 367 Kohka Public Hospital, Koka, Japan; 368 Shiga University of Medical Science Hospital, Otsu, Japan; 369 Otsu City Hospital, Otsu, Japan; 370 Nagahama City Hospital, Nagahama, Japan; 371 Koto Memorial Hospital, Higashiomi, Japan; 372 Yokkaichi Municipal Hospital, Yokkaichi, Japan; 373 Mie University Hospital, Tsu, Japan; 374 Saiseikai Matsusaka General Hospital, Matsusaka, Japan; 375 Ise Red Cross Hospital, Ise, Japan; 376 Shizuoka City Shizuoka Hospital, Shizuoka, Japan; 377 Yaizu City Hospital, Yaizu, Japan; 378 Hamamatsu Rosai Hospital, Hamamatsu, Japan; 379 Hamamatsu Medical Center, Hamamatsu, Japan; 380 Seirei Mikatahara General Hospital, Hamamatsu, Japan; 381 Iwata Municipal General Hospital, Iwata, Japan; 382 NHO Shizuoka Medical Center, Shimizu, Japan; 383 Fuji City General Hospital, Fuji, Japan; 384 Shizuoka Childrens Hospital, Shizuoka, Japan; 385 Gifu Municipal Hospital, Gihu, Japan; 386 Gifu University Hospital, Gihu, Japan; 387 Gifu Prefectural Tajimi Hospital, Tajimi, Japan; 388 Toki General Hospital, Toki, Japan; 389 Saku Central Hospital, Saku, Japan; 390 Aizawa Hospital, Matsumoto, Japan; 391 Nagano Municipal Hospital, Nagano, Japan; 392 Shinonoi General Hospital, Nagano, Japan; 393 Suwa Central Hospital, Chino, Japan; 394 Iida Municipal Hospital, Iida, Japan; 395 Showa Inan General Hospital, Komagane, Japan; 396 Japanese Red Cross Society Azumino Hospital, Azumino, Japan; 397 Azumi General Hospital, Kitaazumi, Japan; 398 NHO Shinshu Ueda Medical Center, Ueda, Japan; 399 Shimane Prefectural Central Hospital, Izumo, Japan; 400 Yasugi Municipal Hospital, Yasugi, Japan; 401 NHO Hamada Medical Center, Hamada, Japan; 402 NHO Okayama Medical Center, Okayama, Japan; 403 Okayama Kyokuto Hospital, Okayama, Japan; 404 Okayama City Hospital, Okayama, Japan; 405 Okayama University Hospital, Okayama, Japan; 406 Kawasaki Medical School Hospital, Kurashiki, Japan; 407 Kurashiki Central Hospital, Kurashiki, Japan; 408 Kurashiki Heisei Hospital, Kurashiki, Japan; 409 Tsuyama Chuo Hospital, Tsuyama, Japan; 410 Okayama Kyoritsu General Hospital, Okayama, Japan; 411 Kasaoka Daiichi Hospital, Kasaoka, Japan; 412 JCHO Tokuyama Central Hospital, Shunan, Japan; 413 Yamaguchi Prefectural Grand Medical Center, Hofu, Japan; 414 Yamaguchi Red Cross Hospital, Yamaguchi, Japan; 415 Yamaguchi University Hospital, Ube, Japan; 416 Ube-Kohsan Central Hospital, Ube, Japan; 417 NHO Kanmon Medical Center, Shimonoseki, Japan; 418 Shimonoseki City Hospital, Shimonoseki, Japan; 419 Hiroshima Red Cross Hospital and Atomic Bomb Survivors Hospital, Hiroshima, Japan; 420 Suiseikai Kajikawa Hospital, Hiroshima, Japan; 421 Hiroshima University Hospital, Hiroshima, Japan; 422 Hiroshima Prefectural Hospital, Hiroshima, Japan; 423 Araki Neurosurgical Hospital, Hiroshima, Japan; 424 Mazda Hospital, Aki, Japan; 425 Chugoku Rosai Hospital, Kure, Japan; 426 Kohsei General Hospital, Mihara, Japan; 427 Mitsugi General Hospital, Onomichi, Japan; 428 Miyoshi Central Hospital, Miyoshi, Japan; 429 Tokushima University Hospital, Tokushima, Japan; 430 Tokushima Prefecture Naruto Hospital, Naruto, Japan; 431 Tokushima Prefectural Kaifu Hospital, Kaifu, Japan; 432 Kansai Medical University Hospital, Hirakata, Japan; 433 Kyushu Rosai Hospital, Kitakyushu, Japan; 434 Teinekeijinkai Hospital, Sapporo, Japan; 435 Sapporo Azabu Neurosurgical Hospital, Sapporo, Japan; 436 Hakodate Central General Hospital, Hakodate, Japan; 437 Otaru Chuo Hospital, Otaru, Japan; 438 NHO Hokkaido Medical Center, Sapporo, Japan; 439 Sapporo Medical University Hospital, Sapporo, Japan; 440 Hakodate Municipal Hospital, Hakodate, Japan; 441 Sapporoteishinkai Hospital, Sapporo, Japan; 442 Shinsapporo Neurosurgical Hospital, Sapporo, Japan; 443 Sapporo Shiroishi Memorial Hospital, Sapporo, Japan; 444 Sendai East Neurosurgical Hospital, Sendai, Japan; 445 JA Akita Kouseiren Oomagarikousei Medical Center, Daisen, Japan; 446 Noshiro Kosei Medical Center, Noshiro, Japan; 447 Iwate Prefectural Kuji Hospital, Kuji, Japan; 448 Tohoku University Hospital, Sendai, Japan; 449 Aomori Prefectural Central Hospital, Aomori, Japan; 450 Iwate Prefectural Central Hospital, Morioka, Japan; 451 NHO Sendai Medical Center, Sendai, Japan; 452 Saitama Red Cross Hospital, Saitama, Japan; 453 Ishinomaki Red Cross Hospital, Ishinomaki, Japan; 454 Osaki Citizen Hospital, Osaki, Japan; 455 Yamagata Saisei Hospital, Yamagata, Japan; 456 Ohta Nishinouchi Hospital, Koriyama, Japan; 457 Tokyo Metropolitan Childrens Medical Center, Fuchu, Japan; 458 Mito Kyodo General Hospital, Mito, Japan; 459 Chiba Rosai Hospital, Ichihara, Japan; 460 Chiba Cancer Center, Chiba, Japan; 461 Chiba Childrens Hospital, Chiba, Japan; 462 Secomedic Hospital, Funabashi, Japan; 463 Saiseikai Kawaguchi General Hospital, Kawaguchi, Japan; 464 Juntendo Tokyo Koto Geriatric Medical Center, Tokyo, Japan; 465 Keio University Hospital, Tokyo, Japan; 466 Jinmeikai Akiyama Neurosurgical Hospital, Yokohama, Japan; 467 Tokyo Dental College Ichikawa General Hospital, Ichikawa, Japan; 468 Mitsuwadai General Hospital, Chiba, Japan; 469 Ebina General Hospital, Ebina, Japan; 470 Tokyo Metropolitan Health and Medical Treatment Corporation Ohkubo Hospital, Tokyo, Japan; 471 Itabashi Chuo Medical Center, Tokyo, Japan; 472 Ome Municipal General Hospital, Ome, Japan; 473 Tokyo Metropolitan Tama Medical Center, Fuchu, Japan; 474 Teraoka Memorial Hospital, Fukuyama, Japan; 475 Kitasato University Hospital, Sagamihara, Japan; 476 Yokohama Asahi Chuo General Hospital, Yokohama, Japan; 477 Yamagata Prefectural Central Hospital, Yamagata, Japan; 478 Japanese Red Cross Akita Hospital, Akita, Japan; 479 Shinshu University Hospital, Matsumoto, Japan; 480 Kobayashi Neurosurgical Neurological Hospital, Ueda, Japan; 481 Komoro Kosei General Hospital, Komoro, Japan; 482 Nagano Childrens Hospital, Azumino, Japan; 483 Ina Central Hospital, Ina, Japan; 484 Toyama University Hospital, Toyama, Japan; 485 Noto General Hospital, Nanao, Japan; 486 Keiju Medical Center, Nanao, Japan; 487 Toyohashi Municipal Hospital, Toyohashi, Japan; 488 Chubu Rousai Hospital, Nagoya, Japan; 489 Kamiiida Daiichi General Hospital, Nagoya, Japan; 490 Nagoya Central Hospital, Nagoya, Japan; 491 Nagoya City University Hospital, Nagoya, Japan; 492 Nagoya City East Medical Center, Nagoya, Japan; 493 Chutoen General Medical Center, Kakegawa, Japan; 494 Japanese Red Cross Takayama Hospital, Takayama, Japan; 495 Daiyukai General Hospital, Ichinomiya, Japan; 496 Seikeikai Hospital, Sakai, Japan; 497 Higashiosaka City Medical Center, Higashiosaka, Japan; 498 Kyoto University Hospital, Kyoto, Japan; 499 Tenri Hospital, Tenri, Japan; 500 Kishiwada City Hospital, Kishiwada, Japan; 501 Otsu Red Cross Hospital, Otsu, Japan; 502 Rakuwakai Otowa Hospital, Kyoto, Japan; 503 Higashisumiyoshi Morimoto Hospital, Osaka, Japan; 504 Daiichitowakai Hospital, Takatsuki, Japan; 505 Kano Hospital, Osaka, Japan; 506 Moriguchi-Ikuno Memorial Hospital, Moriguchi, Japan; 507 Ishikiriseiki Hospital, Higashiosaka, Japan; 508 Osaka Medical Center for Cancer and Cardiovascular Diseases, Osaka, Japan; 509 Rinku General Medical Center, Izumisano, Japan; 510 Hyogo College of Medicine, Nishinomiya, Japan; 511 Akashi City Hospital, Akashi, Japan; 512 Hyogo Prefectural Kakogawa Medical Center, Kakogawa, Japan; 513 Tokushima Red Cross Hospital, Komatsushima, Japan; 514 NHO Shikoku Medical Center for Children and Adults, Zentsuji, Japan; 515 NHO Iwakuni Clinical Center, Iwakuni, Japan; 516 Hiroshima City Hiroshima Citizens Hospital, Hiroshima, Japan; 517 Okayama Saiseikai General Hospital, Okayama, Japan; 518 Fukuyama City Hospital, Fukuyama, Japan; 519 Onomichi Municipal Hospital, Onomichi, Japan; 520 Kaneda Hospital, Maniwa, Japan; 521 Higashihiroshima Medical Center, Higashihiroshima, Japan; 522 Tottori Municipal Hospital, Tottori, Japan; 523 Shuto General Hospital, Yanai, Japan; 524 Hospital of the University of Occupational and Environmental Health, Kitakyushu, Japan; 525 Munakata Suikokai General Hospital, Fukutsu, Japan; 526 JCHO Kyushu Hospital, Kitakyushu, Japan; 527 Kyushu Central Hospital of the Mutual Aid Association of Public School Teachers, Fukuoka, Japan; 528 Harasanshin Hospital, Fukuoka, Japan; 529 Japanese Red Cross Fukuoka Hospital, Fukuoka, Japan; 530 Hakujyuji Hospital, Fukuoka, Japan; 531 Saga University Hospital, Saga, Japan; 532 St. Marys Hospital, Kurume, Japan; 533 Tobata Kyoritsu Hospital, Kitakyushu, Japan; 534 JCHO Hitoyoshi Medical Center, Hitoyoshi, Japan; 535 Oitaken Koseiren Tsurumi Hospital, Beppu, Japan; 536 Sanseikai Kanemaru Neurosurgery Hospitai, Miyazaki, Japan; 537 Atsuchi Neurosurgical Hospital, Kagoshima, Japan; 538 NHO Kagoshima Medical Center, Kagoshima, Japan; 539 Okinawa Red Cross Hospital, Naha, Japan; 540 Asahi General Hospital, Asahi, Japan; 541 Kobe City Medical Center General Hospital, Kobe, Japan; 542 Yoshida Hospital, Kobe, Japan; 543 Wakkanai Teishinkai Hospital, Wakkanai, Japan; 544 Southern Tohoku General Hospital, Iwanuma, Japan; 545 Tokyo General Hospital, Tokyo, Japan; 546 St.Lukes International Hospital, Tokyo, Japan; 547 Chiba Tokushukai Hospital, Funabashi, Japan; 548 Nozaki Tokushukai Hospital, Daito, Japan; 549 Fukuoka University Chikushi Hospital, Chikushino, Japan; 550 Fukuoka Wajiro Hospital, Fukuoka, Japan; 551 Shintakeo Hospital, Takeo, Japan; 552 Tokyo Metropolitan Geriatric Hospital and Institute of Gerontology, Tokyo, Japan; 553 Kansai Electric Power Hospital, Osaka, Japan; 554 Tomakomaihigashi Hospital, Tomakomai, Japan; 555 Date Red Cross Hospital, Date, Japan; 556 Hirosaki Stroke and Rehabilitation Center, Hirosaki, Japan; 557 Kitakami Saiseikai Hospital, Kitakami, Japan; 558 Izumi Hospital, Sendai, Japan; 559 Shin-Oyama City Hospital, Oyama, Japan; 560 Kamagaya General Hospital, Kamagaya, Japan; 561 Nissan Tamagawa Hospital, Tokyo, Japan; 562 Higashitotsuka Memorial Hospital, Yokohama, Japan; 563 Kaetsu Hospital, Niigata, Japan; 564 NHO Niigata Hospital, Kashiwazaki, Japan; 565 Toyama Red Cross Hospital, Toyama, Japan; 566 Maruko Central Hospital, Ueda, Japan; 567 Okaya City Hospital, Okaya, Japan; 568 Kenwakai Hospital, Iida, Japan; 569 Suwa Red Cross Hospital, Suwa, Japan; 570 Kyoto Katsura Hospital, Kyoto, Japan; 571 Shimizu Hospital, Kyoto, Japan; 572 Mimihara General Hospital, Sakai, Japan; 573 Osaka Saiseikai Ibaraki Hospital, Ibaraki, Japan; 574 Kobe Ekisaikai Hospital, Kobe, Japan; 575 Kita-Harima Medical Center, Ono, Japan; 576 Fujii Masao Memorial Hospital, Kurayoshi, Japan; 577 HITO Medical Center, Shikokuchuo, Japan; 578 Chidoribashi Hospital, Fukuoka, Japan; 579 Souseikai Shin Yoshizuka Hospital, Fukuoka, Japan; 580 Miyake Neurosurgical Hospital, Iizuka, Japan; 581 Kitakyushu General Hospital, Kitakyushu, Japan; 582 JCHO Isahaya General Hospital, Isahaya, Japan; 583 Nakatsu Municipal Hospital, Nakatsu, Japan; 584 Nakatsu Neurosurgical Hospital, Nakatsu, Japan; 585 Tomishiro Central Hospital, Tomigusuku, Japan; 586 Otaru General Hospital, Otaru, Japan; 587 Shuuwa General Hospital, Kasukabe, Japan; 588 Fukuchiyama City Hospital, Fukuchiyama, Japan; 589 Saku Central Hospital Advanced Care Center, Saku, Japan; 590 Inazawa Municipal Hospital, Inazawa, Japan; 591 Nishitokyo Central General Hospital, Nishitokyo, Japan; 592 Koyama Memorial Hospital, Kashima, Japan; 593 Shinwakai Yachiyo Hospital, Anjo, Japan; 594 Ainomiyako Neurosurgery Hospital, Osaka, Japan; 595 Ogaki Tokushukai Hospital, Ogaki, Japan; 596 Hamamatsu City Rehabilitation Hospital, Hamamatsu, Japan; 597 Saiseikai Futsukaichi Hospital, Achikushino, Japan; 598 Ashiya Municipal Hospital, Ashiya, Japan; 599 Kyoritsu Hospital, Kawanishi, Japan

**Keywords:** Cerebrovascular disorders, Stroke

## Abstract

To determine whether increasing thrombectomy-capable hospitals with moderate comprehensive stroke center (CSC) capabilities is a valid alternative to centralization of those with high CSC capabilities. This retrospective, nationwide, observational study used data from the J-ASPECT database linked to national emergency medical service (EMS) records, captured during 2013–2016. We compared the influence of mechanical thrombectomy (MT) use, the CSC score, and the total EMS response time on the modified Rankin Scale score at discharge among patients with acute ischemic stroke transported by ambulance, in phases I (2013–2014, 1461 patients) and II (2015–2016, 3259 patients). We used ordinal logistic regression analyses to analyze outcomes. From phase I to II, MTs increased from 2.7 to 5.5%, and full-time endovascular physicians per hospital decreased. The CSC score and EMS response time remained unchanged. In phase I, higher CSC scores were associated with better outcomes (1-point increase, odds ratio [95% confidence interval]: 0.951 [0.915–0.989]) and longer EMS response time was associated with worse outcomes (1-min increase, 1.007 [1.001–1.013]). In phase II, neither influenced the outcomes. During the transitional shortage of thrombectomy-capable hospitals, increasing hospitals with moderate CSC scores may increase nationwide access to MT, improving outcomes.

## Introduction

Mechanical thrombectomy (MT) is the standard of care for patients with large vessel occlusion (LVO) related to acute ischemic stroke (AIS), but only approximately 3% of patients with AIS underwent MT in the US and Japan in 2016 and 2015, respectively^1,2^ patients with AIS and LVO could benefit from direct transportation to intervention centers; however, patients with no LVO need rapid intravenous thrombolysis at the nearest center^3,4^. Patients with AIS admitted directly to comprehensive stroke centers (CSCs) with endovascular treatment capacities may have better outcomes than those receiving drip-and-ship treatment^3^, but timely MT may be impeded by the distance that patients need to travel to thrombectomy-capable hospitals. Is there a valid alternative to regional centralization of MT to CSCs with high CSC capabilities to achieve better AIS patient outcomes^5^?

The existence of a single national emergency medical service (EMS) system in Japan provides a unique opportunity to conduct nationwide studies to examine the influence of prehospital time and hospital characteristics on clinical outcomes using linked data^6^.

Using data from the J-ASPECT (Nationwide survey of Acute Stroke care capacity for Proper dEsignation of Comprehensive stroke cenTer in Japan) study—a hospital-based, Japan-wide stroke registry^7,8^—we examined whether increasing thrombectomy-capable hospitals with moderate CSC capabilities^2,9^ is a valid alternative for regional centralization of MT to CSCs with high CSC capabilities^3,5^. Accordingly, we compared the influence of CSC capabilities and prehospital time on outcomes of MT for patients with AIS during the transitional phase before and after the pivotal trials^10^.

## Methods

### Ethics statement

To maintain confidentiality, we used deidentified databases, for which it would have been impracticable to obtain informed consent. The study was approved by the National Cerebral and Cardiovascular Center Institutional Review Board (M29-161-4, M29-161-3, and M29-088-3), which waived the requirement for individual informed consent. We reiterate that all methods were performed in accordance with the relevant guidelines and regulations.

### J-ASPECT study

Participation of hospitals in the J-ASPECT study was voluntary, and the study was performed in collaboration with the Japan Neurosurgical Society (JNS) and Japan Stroke Society (JSS)^2,7,8,11^. This study consisted of two projects: (1) an institutional survey to assess the CSC capabilities of the hospitals (Table 1)^9,12^ and (2) a retrospective cohort study using the nationwide Diagnosis Procedure Combination (DPC) inpatient database^2,7,8,11^. Briefly, the DPC is a mixed-case classification system linked with a lump-sum payment system that was launched in 2002 by the Ministry of Health, Labor, and Welfare of Japan. By 2015, the DPC system had been adopted by an estimated 1580 acute care hospitals, representing approximately half of all Japanese hospital beds and encompassing a wide variety of centers, including rural and urban, academic and non-academic, and small and large hospitals^7,13^. An increasing number of acute stroke cases in Japan are being registered in the J-ASPECT study each year^14^, with approximately 1,090,000 cases registered as of October 2020.

**Table 1:** Comprehensive stroke center score components and items.

Item No	Components	Items
1	Personnel*	Neurologists
2		Neurosurgeons
3		Endovascular physicians
4		Emergency medicine
5		Physical medicine and rehabilitation
6		Rehabilitation therapy
7		Stroke nurses
8	Diagnostic (24/7)	Computed tomography
9		Magnetic resonance imaging with diffusion
10		Digital cerebral angiography
11		Computed tomography angiography
12		Carotid duplex ultrasound
13		Transcranial Doppler
14	Specific expertise	Carotid endarterectomy
15		Clipping of intracranial aneurysm
16		Hematoma removal/draining
17		Coiling of intracranial aneurysm
18		Intra-arterial reperfusion therapy
19	Infrastructure	Stroke unit
20		Intensive care unit
21		Operating room staffed 24/7
22		Interventional services coverage 24/7
23		Stroke registry
24	Education	Community education
25		Professional education

*Availability of full-time, board-certified personnel.

### Assessment of facility capabilities using the institutional survey

We sent out questionnaires in 2014 to assess the CSC capabilities of the facilities using 25 items recommended by the Brain Attack Coalition to the training institutions of the JNS and JSS^7,9^. The items are classified into five categories, as follows: personnel, diagnostic, specific expertise, infrastructure, and education (Table 1)^7,9^. A score of 1 point was assigned for each recommended item met by the hospital, yielding a total CSC score of up to 25. The content, development, and predictive validities of this scoring system for measuring CSC capabilities have been reported^7,9^. Our study group previously showed that the CSC score categorized into either quintiles or quartiles is associated with short-term clinical outcomes of patients with ischemic and hemorrhagic stroke including those who received surgical and endovascular treatment for stroke. In this study, we categorized the participating thrombectomy-capable hospitals based on the CSC scores into low (< 17), moderate (17–21) and high (> 21).

### Study design

We conducted a retrospective observational study using data from the J-ASPECT stroke database (specifically those that could be linked to the national EMS records) captured between January 2013 and December 2016. Since the JSS, JNS, and Japanese Society for Neuroendovascular Therapy (JSNET) acted quickly to revise the clinical practice guidelines for MT use in response to the publication of the pivotal trials^10^, we divided the study period into phases I (2013–2014) and II (2015–2016). After publishing the results of the pivotal trials, the American Heart Association/American Stroke Association (AHA/ASA) updated the clinical guidelines for the endovascular treatment of acute ischemic stroke in 2015^15^. In this guideline, patients should receive endovascular therapy with a stent retriever if they meet all of the criteria. In response to this, the relevant Japanese Society updated guidelines for mechanical thrombectomy^16^. Since the publication of these guidelines^15^^.^^16^, mechanical thrombectomy has been considered as standard treatment for large-vessel occlusion acute ischemic stroke regardless of geographical locations. Using the J-ASPECT DPC database, we extracted data of 303,719 patients with AIS diagnosed based on the diagnostic criteria of the International Classification of Diseases, 10th Revision (ICD-10) diagnosis code I63. Of those, we included 163,292 records of patients directly transported to facilities by ambulance, in the record linkage analysis. Patients’ consciousness level at admission was measured using the Japan Coma Scale (JCS) for stroke severity (Supplementary Table S1)^7,17^.

In this study, we defined thrombectomy-capable hospitals as those where MT was performed at least once in each phase. Using government data from 2015, we further categorized hospitals into three classes according to the population density in the regions that they serve, as follows: < 300 persons/km^2^, 300–1000 persons/km^2^, and > 1000 persons/km^2^.^18^.

We obtained permission to use all EMS data for 2013 and 2016 from the Fire and Disaster Management Agency of the Ministry of Internal Affairs and Communications^6^. Almost 99% of 771 EMS departments all over Japan participated in this study, which yielded 21,327,841 patient records. Using this database, we calculated the total EMS response time (i.e., from receipt of the emergency call to arrival at the hospital), which includes EMS response time, on-scene time, and transport time.

### Data linkage and participant selection

We used one-to-one probabilistic linkage using the relink module in Stata 15.1 to match the J-ASPECT study and EMS records using the following five linkage points: date of incident/admission, hospital code, prefectures code, age, and sex^19,20^. A participant was included only if there was a linkage across all these characteristics. After linkage, we identified patients with AIS and excluded those who (1) were younger than 18 years; (2) had been transferred from another facility; or (3) had no valid timestamps, had duplicate cases, or had missing care outcomes. We used information from insurance claims to constitute the MT group by identifying all patients treated with any kind of device, including Merci (Concentric Medical, Mountain View, CA, US), Penumbra (Penumbra, Alameda, CA, US), Trevo ProVue (Stryker, Kalamazoo, MI, US), and Solitaire-FR (Covidien, Irvine, CA, US).

### Study outcomes

We assessed outcomes using the modified Rankin Scale (mRS) score at discharge.

### Statistical analyses

To compare patient characteristics and outcomes between phases I and II, we used *t*-tests for normally distributed continuous variables and Mann–Whitney U tests for non-normally distributed continuous variables. We compared categorical variables using Fisher’s exact or chi-square tests. After linking the records, we used multiple imputations to handle missing data points regarding the CSC scores, body mass index (BMI), and smoking history^21^. We used ordinal logistic regression analyses to investigate the associations of total EMS response time and CSC score with the mRS score at discharge, while adjusting for confounding patient-related (age, sex, stroke severity, comorbidities, BMI, smoking history, and total EMS response time) and hospital-related (CSC score^9^ and geographical category^18^) factors. We performed all analyses using JMP Statistical Software version 12 (SAS Institute Inc., Cary, NC, US) and Stata version 15.1 (Stata Corp., College Station, TX, US). A *p*-value < 0.05 was considered significant.

## Results

### Probabilistic record linkage

**Figure 1:**
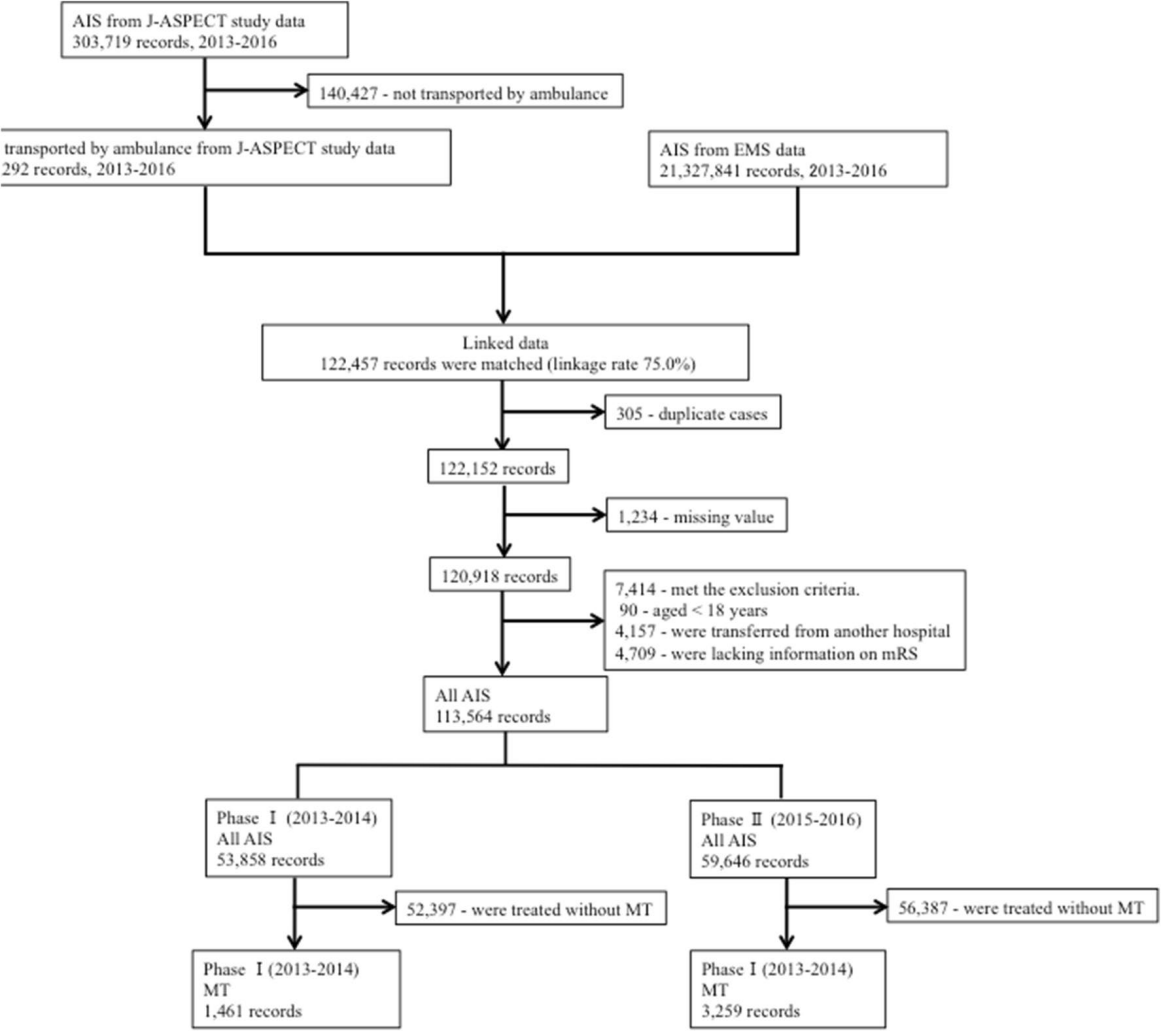
Flow diagram of patients. *AIS* acute ischemic stroke; *EMS* emergency medical services; *mRS* modified Rankin Scale.

We were able to link 122,457 patients with AIS using the J-ASPECT and EMS data (linkage rate of 75.0%). After implementing the exclusion criteria, we included 113,564 records of patients with AIS in the final analyses (Fig. 1). Among them, 1461 patients with AIS received MT from 170 hospitals in phase I and 3259 patients received MT from 206 hospitals in phase II. From phase I to II, the proportion of patients who received MT according to population density remained unchanged (Table 2).

**Table 2:** Patient characteristics, EMS time metrics, treatment, and outcomes in all AIS and MT groups.

	All AIS		p	MT group		p
	Phase I	Phase II		Phase I	Phase II	
	(n = 53,858)	(n = 59,646)		(n = 1,461)	(n = 3,259)	
Age (years), mean (SD)	75.7 (12.1)	76.0 (12.1)	< .001	73.4 (11.3)	74.7 (11.6)	< .001
Men, n (%)	30,850 (57.3)	34,163 (57.3)	0.989	839 (57.4)	1,839 (56.4)	0.522
JCS score at admission, n (%)			< .001			0.010
Alert	18,726 (34.8)	21,070 (35.3)		92 (6.3)	282 (8.7)	
Awake	24,137 (44.8)	26,988 (45.3)		633 (43.3)	1,450 (44.5)	
Arousable	7,021 (13.0)	7,700 (12.9)		485 (33.2)	1,044 (32.0)	
Unarousable	3,974 (7.3)	3,888 (6.5)		251 (17.2)	483 (14.8)	
Baseline mRS, median, (min, max)	1 (0, 3)	0 (0, 2)	< .001	0 (0, 2)	0 (0, 2)	0.552
**Comorbidities**						
Hypertension, n (%)	32,355 (60.1)	37,437 (62.8)	< .001	1,170 (80.1)	2,642 (81.1)	0.427
Hyperlipidemia, n (%)	17,500 (32.5)	20,370 (34.2)	< .001	364 (24.9)	939 (28.8)	0.006
Diabetes mellitus, n (%)	11,474 (21.3)	12,885 (21.6)	0.222	326 (22.3)	698 (21.4)	0.490
Atrial fibrillation, n (%)	13,746 (25.5)	16,439 (27.6)	< .001	717 (49.1)	1,738 (53.3)	0.007
**EMS time in min, median (IQR)**						
Response time	7 (6, 9)	7 (6, 10)	< .001	7 (6, 9)	7 (6, 9)	0.168
On-scene time	13 (10, 18)	13 (10, 18)	< .001	14 (10, 18)	13 (10, 17)	0.036
Transport time	10 (6, 16)	10 (7, 16)	< .001	10 (6, 16)	10 (6, 16)	0.858
Total EMS response time	33 (26, 41)	33 (27, 42)	< .001	33 (26, 41)	32 (26, 41)	0.404
**Outcome (mRS score)**						
0	5,790 (10.8)	6,855 (11.5)	< .001	87 (6.0)	296 (9.1)	< .001
1	10,050 (18.7)	11,339 (19.0)		150 (10.3)	398 (12.2)	
2	7,811 (14.5)	8,851 (14.8)		182 (12.5)	419 (12.9)	
3	6,530 (12.1)	7,463 (12.5)		142 (9.7)	380 (11.7)	
4	11,508 (21.4)	12,840 (21.5)		397 (27.2)	785 (24.1)	
5	7,685 (14.3)	8,154 (13.7)		287 (19.6)	638 (20.0)	
6	4,484 (8.3)	4,144 (6.9)		216 (14.8)	343 (10.5)	
**Treatment**						
rtPA administration	5,452 (10.1)	6,868 (11.5)	< .001	782 (53.5)	1,763 (54.1)	0.716
MT	1,461 (2.7)	3,529 (5.5)	< .001	1,461 (100)	3,529 (100)	
**Device, n (%)**						
Stent retriever				433 (29.6)	2,275 (69.8)	< .001
Penumbra				1,079 (73.9)	2,124 (65.2)	< .001
Merci				196 (13.4)	6 (0.2)	< .001
Population density, person/km^2^			< .001			0.101
< 300	14,762 (27.9)	15,148 (25.7)		303 (20.7)	702 (21.6)	
300–1000	15,489 (29.3)	18,020 (30.6)		409 (28.0)	988 (30.4)	
> 1000	22,685 (42.9)	15,761 (43.7)		748 (51.2)	1,556 (47.9)	

*AIS* acute ischemic stroke; *EMS* emergency medical service; *IQR* interquartile range; *JCS* Japan Coma Scale; *mRS* modified Rankin Scale; *MT* mechanical thrombectomy; *rt-PA* intravenous recombinant tissue-plasminogen activator infusion; *SD* standard deviation.

### Comparison of patient characteristics between phases I and II

In the MT group, patients in phase II were significantly older, experienced less severe strokes, had a higher frequency of hyperlipidemia and atrial fibrillation, were treated more frequently with MT (5.5% vs. 2.7%), and had better in-hospital outcomes (30-day mortality 10.5% vs. 14.8%) than those in phase I (Table 2). From phase I to II, the use of stent retrievers significantly increased from 29.6 to 69.8%, whereas the use of aspiration catheters (Penumbra) and Merci retrievers significantly decreased from 73.9 to 65.2% and from 13.4 to 0.2%, respectively. The proportion of patients who received recombinant tissue-plasminogen activator (rt-PA) administration before MT remained the same between both phases. We observed similar findings for the characteristics of all patients with AIS except for a higher frequency of hypertension, a higher baseline mRS score, and a lower rt-PA administration score in phase I.

### Prehospital time of the participating hospitals in each phase

In the MT groups, the total EMS response time remained almost the same between phase I and II, despite a shorter on-scene time in phase II (Table 2). We observed no clinically meaningful differences in prehospital time metrics in all AIS patients between phases. Notably, the transport time in the MT groups remained the same from phase I to II and comparable with those of all patients with AIS in each phase.

### CSC capabilities of the participating hospitals in each phase

The CSC capabilities based on hospital characteristics are summarized in Table 3. In phases I and II, the percentages of missing CSC score data in the MT groups were 6.0% and 14.7%, respectively. Among all participating hospitals, the proportion of thrombectomy-capable hospitals increased from 45.6 to 58.2% from phase I to II. The median CSC scores of all participating hospitals and thrombectomy-capable hospitals remained unchanged between phases. Although there were no between-phase differences in the CSC scores for thrombectomy-capable hospitals, the difference in the median CSC scores between all participating and thrombectomy-capable hospitals in phase II became smaller than that in phase I (17 vs. 18 in phase I, 18 vs. 19 in phase II). Among the 25 items used to assess CSC capabilities, we observed no between-phase differences in availability in all participating hospitals; however, in the thrombectomy-capable hospitals, availability of the items related to endovascular treatment such as full-time, board-certified endovascular physicians and intra-arterial reperfusion therapy significantly decreased in phase II (*p* = 0.05, < 0.03) (Table 3).

**Table 3:** CSC capabilities based on hospital characteristics in all groups.

	All participating hospitals		p	Thrombectomy-capable hospitals		p
	Phase I	Phase II		Phase I	Phase II	
	(n = 373)	(n = 354)		(n = 170)	(n = 206)	
Neurologists	210 (56.3)	200 (56.5)	0.976	104 (61.2)	124 (60.2)	0.846
Neurosurgeons	364 (97.6)	347 (98.0)	0.702	168 (98.8)	204 (99.0)	0.847
Endovascular physicians	216 (57.9)	212 (59.9)	0.600	151 (88.8)	168 (81.6)	**0.050**
Emergency medicine	143 (38.3)	140 (39.6)	0.787	73 (42.9)	89 (43.2)	0.959
Physical medicine and rehabilitation	104 (27.9)	95 (26.8)	0.675	43 (25.3)	57 (27.7)	0.604
Rehabilitation therapy	372 (99.7)	353 (99.7)	0.966	169 (99.4)	205 (99.5)	0.892
Stroke rehabilitation nurses	116 (31.1)	111 (31.4)	0.990	68 (40.0)	81 (39.3)	0.893
Computed tomography	371 (99.5)	351 (99.2)	0.605	170 (100.0)	206 (100)	-
Magnetic resonance imaging with diffusion	359 (96.2)	340 (96.1)	0.870	169 (99.4)	203 (98.5)	0.414
Digital cerebral angiography	346 (92.8)	331 (93.5)	0.716	168 (98.8)	203 (98.5)	0.814
Computed tomography angiography	352 (94.4)	334 (94.4)	0.969	166 (97.6)	203 (98.5)	0.522
Carotid duplex ultrasound	156 (41.8)	154 (43.5)	0.631	80 (47.1)	104 (50.5)	0.508
Transcranial Doppler	91 (24.4)	92 (26.0)	0.636	54 (31.8)	67 (32.5)	0.875
Carotid endarterectomy	332 (89.0)	316 (89.3)	0.845	163 (95.9)	195 (94.7)	0.581
Clipping of intracranial aneurysm	360 (96.5)	345 (97.5)	0.471	170 (100.0)	205 (99.5)	0.363
Hematoma removal/draining	359 (96.2)	345 (97.5)	0.362	169 (99.4)	205 (99.5)	0.892
Coiling of intracranial aneurysm	248 (66.5)	237 (67.0)	0.895	162 (95.3)	180 (87.4)	**0.008**
Intra-arterial reperfusion therapy	290 (77.7)	281 (79.4)	0.569	167 (98.2)	193 (93.7)	**0.030**
Stroke unit	144 (38.6)	138 (39.0)	0.968	82 (48.2)	97 (47.1)	0.825
Intensive care unit	282 (75.6)	265 (74.9)	0.834	136 (80.0)	168 (81.6)	0.703
Operating room staffed 24/7	250 (67.0)	240 (67.8)	0.823	145 (85.3)	173 (84.0)	0.726
Interventional services coverage 24/7	241 (64.6)	237 (67.0)	0.507	158 (92.9)	180 (87.4)	0.075
Stroke registry	198 (53.1)	195 (55.1)	0.505	102 (60.0)	131 (63.6)	0.475
Community education	107 (28.7)	101 (28.5)	0.953	63 (37.1)	71 (34.5)	0.601
Professional education	231 (62.1)	225 (63.6)	0.656	123 (72.4)	150 (72.8)	0.920
CSC score	17 (14, 20)	18 (14, 20)	0.725	19 (17, 21)	19 (17, 21)	0.447
Population density, person/km^2^			0.888			0.887
< 300	108 (29.9)	105 (30.4)		47 (27.8)	54 (26.5)	
300–1000	107 (29.6)	107 (30.9)		50 (29.6)	65 (31.9)	
> 1000	146 (40.4)	134 (38.7)		72 (42.6)	85 (41.7)	

*CSC* comprehensive stroke center; * implementation of full-time, board-certified personnel. P-values in bold are significant.

### Associations between total EMS response time and clinical outcomes

**Figure 2:**
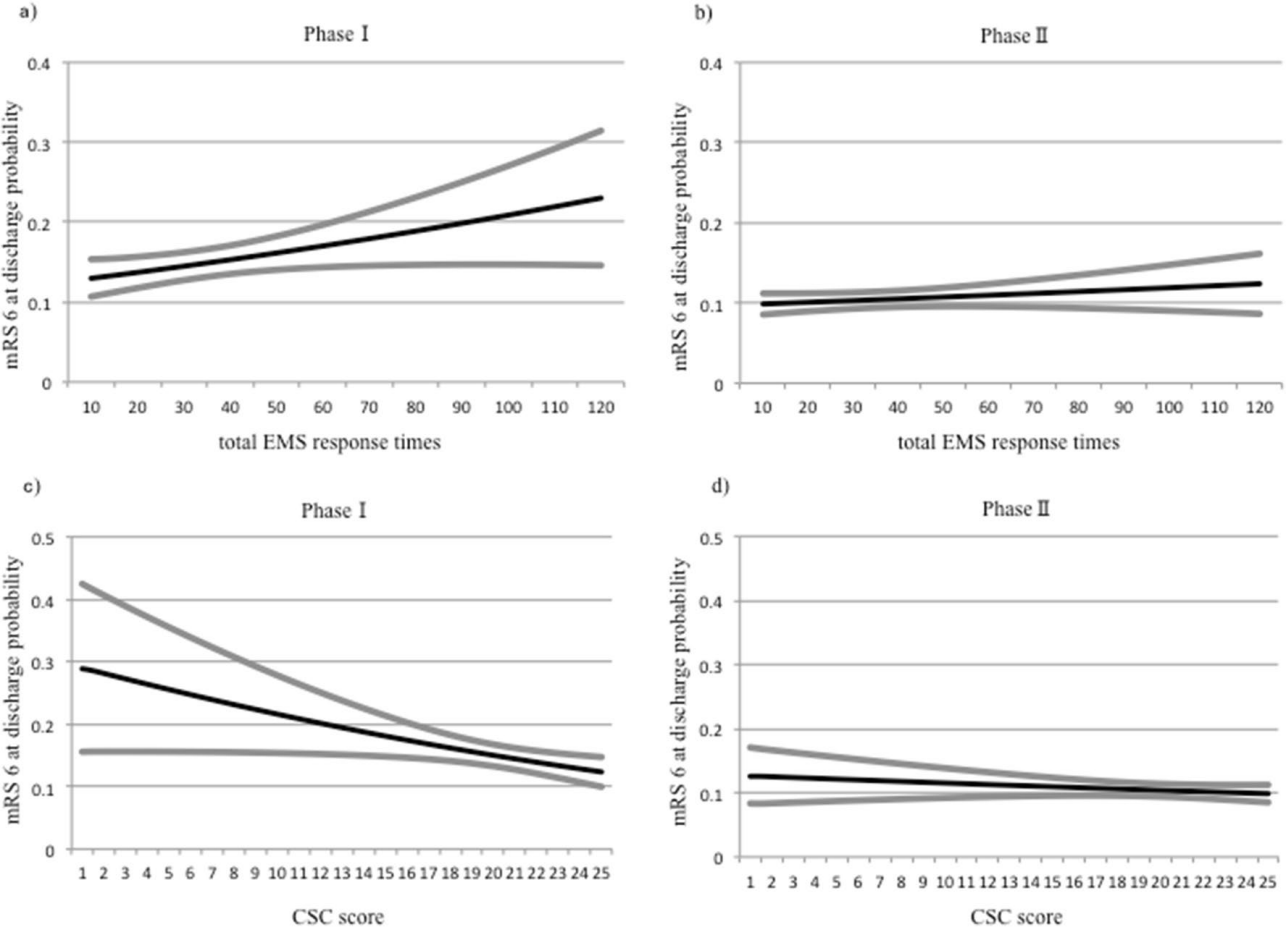
Relationships between the total EMS response time or the CSC scores and probabilities of an mRS score of 6 at discharge (stroke outcomes) in the MT group. Panels (**a**) and (**b**) show the effects of total EMS response time (minutes) on the probabilities of an mRS score of 6 at discharge in phases I and II, respectively, in the MT group. Panels (**c**) and (**d**) show the effects of the CSC scores on the probabilities of an mRS score of 6 at discharge in phases I and II, respectively, in the MT group. *EMS* emergency medical services; *CSC* comprehensive stroke center; *mRS* modified Rankin Scale; *MT* mechanical thrombectomy.

No clinically meaningful differences in the median total EMS response time were observed between all patients with AIS and the MT group in each phase (e.g., 33 vs. 32 min in phase II) (Table 2). In the MT group, a longer total EMS response time was associated with worse outcomes in phase I (odds ratio [OR] for each 1-min increase, 1.007 [CI, 1.001–1.013]); however, this association was not observed in phase II (1.003 [0.998–1.007]). The relationships between the total EMS response time and probabilities of an mRS score of 6 at discharge in phases I and II in the MT groups are shown in Fig. 2a, b.

**Figure 3:**
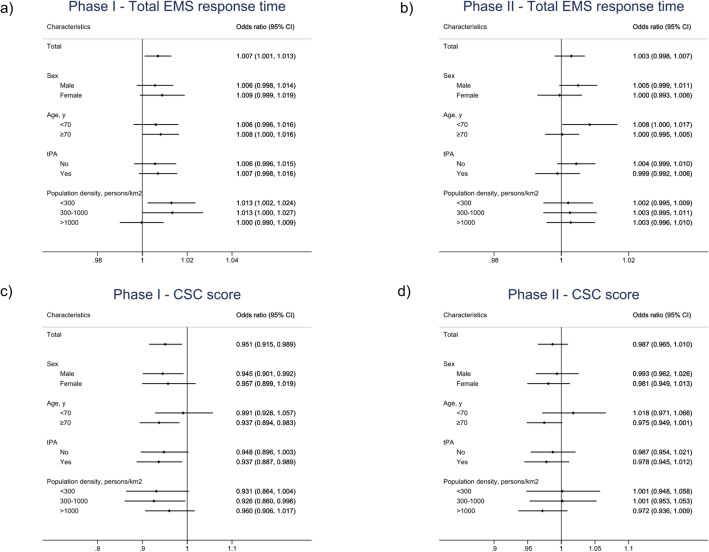
Subgroup analyses of the effect of the total EMS response time and the CSC score on outcomes. The forest plot shows the effect size of a 1-min increase of the total EMS response time on the mRS score at discharge in phases I (panel **a**) and II (panel **b**), respectively, in the MT group, analyzed according to ordinal logistic regression across subgroups. Dots indicate point estimates for the effect of the total EMS response time. The forest plot shows the effect size of a 1-point increase of the CSC score on the mRS score at discharge in phases I (panel **c**) and II (panel **d**), respectively, in the MT group, analyzed according to ordinal logistic regression across subgroups. Dots indicate point estimates for the effect of the CSC score. *EMS* emergency medical services; *CSC* comprehensive stroke center; *mRS* modified Rankin Scale; *MT* mechanical thrombectomy.

Subgroup analyses demonstrated that the effect of total response time on clinical outcomes in phase I was notable for patients aged ≥ 70 years and those who reside in areas with low and intermediate population density (< 300, 300–1000 persons/km^2^; Fig. 3a). This association was only noted for patients aged < 70 years in phase II (Fig. 3b).

### Associations between CSC score and clinical outcomes

In phase I, there was an association between an increase in the CSC score and better outcomes after MT (each 1-point increase, 0.951 [0.915–0.989]). In phase II, however, the association between a higher CSC score and improved outcomes was no longer observed in the MT group (each 1-point increase, 0.987 [0.965–1.010]). The relationships between the CSC score and probabilities of an mRS score of 6 at discharge in phases I and II in the MT groups are shown in Figs. 2c, d.

Subgroup analyses demonstrated that the effect of CSC scores on clinical outcomes in phase I was notable for men, patients aged ≥ 70 years, those who received preceding rt-PA administration, and those who resided in areas with intermediate population density (300–1000 persons/km^2^) (Fig. 3c). No influence of the CSC score on clinical outcomes was observed in any subgroup in phase II (Fig. 3d).

## Discussion

Linking data from the J-ASPECT stroke database from 2013 to 2016 to the national EMS records, we demonstrated the increased use of MT and better clinical outcomes after MT with less initial stroke severity in an increasing number of thrombectomy-capable hospitals following revisions to the clinical practice guidelines for MT in 2015 by the relevant societies in Japan. Although the availability of endovascular physicians in all participating hospitals remained the same during the study period, fewer endovascular physicians were present in thrombectomy-capable hospitals in phase II, probably because of the rapid nationwide increase of such hospitals in response to the abovementioned revisions.

The influence of the CSC capabilities of the thrombectomy-capable hospitals and prehospital time on the clinical outcomes of patients with AIS who received MT in phase I may support the implementation of regional centralization of thrombectomy-capable hospitals^3,5,22^. No such clinical influence was observed in phase II; however, we posit that, in the current transitional period, while there is a relative shortage of thrombectomy-capable hospitals, increasing the amount of hospitals with moderate CSC scores may benefit the populations of countries with geographical conditions similar to those of Japan.

### Temporal changes in patient characteristics

We demonstrated temporal changes in patient characteristics in the MT group, such as a higher age, decreased stroke severity, and better outcomes in phase II, which were consistent with those reported in previous studies of patients with AIS^2,23^. MT use in patients with AIS who were directly transported to a suitable facility via an ambulance in phase I (2.7%) was comparable to the findings from the US hospitals that participated in the Get With The Guidelines-Stroke program (MT use 3.3%)^1^. MT use (5.5%) in patients with AIS in phase II doubled from that in phase I, which may be explained by an increased awareness of the effectiveness of MT and the implementation of thrombectomy-capable hospitals in response to the revised guidelines in Japan^24^.

In contrast, MT use in patients with AIS who were directly transported via an ambulance to a suitable facility in phase II was almost twice the proportion (3.0%) of all patients with AIS who were urgently hospitalized in Japan from April 2010 to March 2016^2^. This is consistent with the findings of previous studies showing that only 60% of urgently hospitalized patients with AIS are transported via an ambulance^7,25^, suggesting the underuse of ambulances for patients with AIS who are possible candidates of MT in Japan.

### Influence of the CSC capabilities and total EMS response time of thrombectomy-capable hospitals on clinical outcomes in phases I and II

The observed influence of CSC capabilities of thrombectomy-capable hospitals on clinical outcomes of patients with AIS who received MT in phase I may support the concept of regional centralization of thrombectomy-capable hospitals^3,5,22^. This is consistent with our previous studies using data before 2015. Therein, we demonstrated that hospitals with higher (vs. lower) CSC capabilities were more likely to have lower in-hospital mortality among patients with AIS and to provide timely rt-PA infusion and MT on a 24-h basis^2,7^. Although the CSC score comprises heterogeneous items of stroke care expertise, it reflects the joint effort of multiple healthcare professionals to manage emergencies^7,9,11,12^.

One major finding of this study was the lack of association between the CSC capabilities and clinical outcomes of MT in phase II. This unexpected finding may be explained by several observations. First, and most notably, despite the similar CSC scores in phases I and II in the thrombectomy-capable hospitals in this study, the availability of specific items related to endovascular therapy (e.g., full-time availability of board-certified endovascular physicians and intra-arterial reperfusion therapy) decreased. This relative shortage was probably because of the rapid increase in hospitals where MT can be performed. In contrast, in all participating hospitals, the availability of those items remained almost the same from phase I to II, which is consistent with the results in our previous study. Therein, we showed that the implementation of six items, mainly related to endovascular therapy, increased > 20% from 2010 to 2018, especially between 2010 and 2014^12^.

Second, even in thrombectomy-capable hospitals with comparable CSC capabilities in phases I and II, there may be a difference in the quality of in-hospital care related to MT, depending on the availability of endovascular physicians^26–29^. For example, thrombectomy-capable hospitals without sufficient in-house endovascular physicians are more likely to make use of those from neighboring hospitals to perform MT, which may increase the onset-to-reperfusion time and worsen the clinical outcomes^26,30^.

Another key finding of this study was that the total EMS response time was not associated with clinical outcomes of MT in phase II, regardless of the level of urbanization. The influence of the total EMS response time on clinical outcomes in the MT group in phase I is in line with previous studies evaluating the effect of onset-to-treatment time on outcomes of patients who received MT^10^. The total median EMS response time (phase I, 33 min; phase II, 32 min) in the MT group remained unchanged between phase I and II and was shorter than that in a US study (36 min)^31^, suggesting that the total EMS response time may not be an effective target in shortening the onset-to-treatment time. However, this may be characteristic of countries, such as Japan, where a greater proportion of the population lives closer to hospitals than that in more expansive countries^18^. In more expansive countries, driving time exceeding 90 min may be more common. Despite this, we believe that our findings may not be unique to Japan^18^ (e.g., 79% of adults in the US reside within 60 min of a hospital that provides acute cardiac therapy^32^).

The influence of the total EMS response time in phase II may be outweighed by other processes involved in the onset-to-treatment time, such as a delay in EMS activation^33^ and the in-hospital workflow before MT^34^. In this study, we did not have information on the time from symptom recognition to the ambulance call or on the in-hospital workflow; therefore, we could not quantify the role of those processes on the outcomes of patients with AIS who received MT^26,35^. A recent study suggested that patients with a lower socioeconomic status may be more likely to delay EMS activation than those with a higher status^33^; however, educational campaigns raising awareness of the signs and symptoms of stroke have had little effect on the actual response to a stroke event^36,37^. In contrast, fast reperfusion is a modifiable factor associated with better clinical outcomes when successful reperfusion is achieved^34,38^. A recent meta-analysis of the pivotal trials that led to the change in guidelines showed that the intermediary outcome, the rate of successful reperfusion, was higher with faster (vs. slower) hospital-arrival-to-groin-puncture time^34^. This nationwide study lends real-world support to the findings of existing literature on the importance of in-hospital workflow to improve clinical outcomes of patients with AIS who receive MT^38,39^.

In the US, AIS care and quality may differ between institutions, with CSCs outperforming primary stroke centers (PSCs) in timely acute reperfusion therapy and risk-adjusted mortality^33^. Recently, we developed the Close The Gap-Stroke (CTGS) program, the first nationwide quality improvement program within the J-ASPECT study; it allows prospective evaluation of the quality of acute stroke care in Japan, using the DPC database and electronic medical records^14,29^. Further studies are necessary to examine the influence of CSC capabilities on performance in terms of quality indicators and clinical outcomes of patients with AIS who received MT after the JSS started to certify PSCs who are able to perform MT.

Our study suggests that equal accessibility to MT remains an urgent unmet need in real-world situations, which may justify the nationwide implementation of thrombectomy-capable hospitals with moderate CSC capabilities since 2015^40^.

In 2019, in Japan, the JSNET started to certify endovascular physicians who are qualified only to perform MT, and the JSS started to certify PSCs that are encouraged to perform MT. Availability of MT in the PSCs is in line with the increasing availability of endovascular treatment at PSCs in the US^40^. The current findings may lend support to this certification policy, as it may assist in equalizing the accessibility to MT.

## Limitations

First, selection bias and unmeasured residual confounders may exist^10^. The participating hospitals in the J-ASPECT study were more likely to commit to quality improvement in stroke care than non-participating hospitals; however, the number of MTs performed in phase I corresponded to approximately 74.4% of those reported in the Japanese Registry of Neuroendovascular Therapy—the official registry of the JSNET^41^. Further, the geographical locations of the thrombectomy-capable and all participating hospitals in this study were comparable with those reported in the previous nationwide study on rt-PA use in Japan^18^. This suggests that the findings may represent the real-world situation in Japan. Second, the DPC database lacks data regarding several important factors, including the National Institutes of Health Stroke Scale (NIHSS) score, time metrics, and imaging results^7,8,13,14^. Thus, we used the JCS score, rather than the NIHSS score, as an index of stroke severity. Nationwide implementation of the CTGS program of the J-ASPECT study may solve this issue. Third, the LVO site was not included in the analysis; however, our recent study showed that approximately 86.4% of patients with AIS underwent MT from January 2013 to December 2015 according to the guidelines^29^. Fourth, we did not examine the effect of CSC capabilities on in-hospital care provision^29,39^. The result of the CTGS, an ongoing nationwide quality improvement initiative in Japan, may answer this question^14,29^. Finally, long-term outcomes (≥ 90 days) after AIS were not evaluated. Further studies are necessary to address these issues.

## Conclusion

In the current transitional period, while there is a relative shortage of thrombectomy-capable hospitals, increasing the number of hospitals with moderate CSC scores may benefit the general Japanese population by equalizing access to MT in response to AIS. Certification of endovascular physicians qualified to perform MT may also promote such accessibility in thrombectomy-capable hospitals.

## Supplementary Information

Supplementary Information.

## Data Availability

We have documented the data, methods, and materials used to conduct the research in this report. The individual patient data are not publicly available owing to the memorandum signed by the directors of the participating hospitals and the principal investigator of the J-ASPECT Study group.
