# Supplementary material for: Influence of hospital capabilities and prehospital time on outcomes of thrombectomy for stroke in Japan from 2013 to 2016
Source: Sci Rep. 2022 Feb 28;12:3252. doi: 10.1038/s41598-022-06074-1 (PMC8885934; doi:10.1038/s41598-022-06074-1)
Supplement: Supplementary file 1 — Supplementary Information. [file 41598_2022_6074_MOESM1_ESM.docx]

**Supplementary Table S1.** Japan Coma Scale for grading impaired consciousness*.

| Grade | Consciousness level |  |
| --- | --- | --- |
| 1-digit code | The patient is awake without any stimuli and is |  |
| 1 | Almost fully conscious |  |
| 2 | Unable to recognize time, place, and person |  |
| 3 | Unable to recall name or date of birth |  |
| 2-digit code | The patient can be aroused (then reverts to the previous state after cessation of stimulation): |  |
| 10 | Easily, by being spoken to (or is responsive with purposeful movements, phrases, or words)† | |
| 20 | With a loud voice or shaking of shoulders (or is almost always responsive to very simple words like yes or no, or movements) | |
| 30 | Only by repeated mechanical stimuli |  |
| 3-digit code | The patient cannot be aroused with any forceful mechanical stimuli, and: |  |
| 100 | Responds with movements to avoid the stimulus |  |
| 200 | Responds with slight movements, including decerebrate and decorticate posture |  |
| 300 | Does not respond at all except for changes in respiratory rhythm |  |

*“R” and “I” were added to the grade to indicate restlessness and incontinence of urine and feces, respectively: for example, 100-R and 30-RI.

†C

**Supplementary Table S2.** List of the J-ASPECT Study Collaborators.

| Hospitals | Responsible persons |
| --- | --- |
| Ageo Central General Hospital | Hidekazu Takahashi |
| Ainomiyako Neurosurgery Hospital | Isao Sasaki |
| Aizawa Hospital | Kazuo Kitazawa |
| Akashi City Hospital | Minoru Saitoh |
| Akita City Hospital | Hitoshi Saito |
| Akita University Hospital | Hiroaki Shimizu |
| Ako City Hospital | Minoru Asahi |
| Almeida Memorial Hospital | Makoto Goda |
| Aomori City Hospital | Atsuhito Takemura |
| Aomori Prefectural Central Hospital | Tatsuya Sasaki |
| Araki Neurosurgical Hospital | Masaaki Shibukawa |
| Arao Municipal Hospital | Isao Fuwa |
| Asahi General Hospital | Saburo Watanabe |
| Ashikaga Red Cross Hospital |  |
| Ashiya Municipal Hospital | Seiko Kataoka |
| Atsuchi Neurosurgical Hospital | Koji Takasaki |
| Ayabe City Hospital | Kouji Shiga |
| Azumi General Hospital | Kensuke Hayashida |
| Baba Memorial Hospital | Hidefuku Gi |
| Bellland General Hospital | Ryunosuke Uranishi |
| Chiba Cancer Center | Toshihiko Iuchi |
| Chiba Cerebral and Cardiovascular Center | Junichi Ono |
| Chiba Children's Hospital | Chiaki Ito |
| Chiba Neurosurgical Clinic | Kenji Wakui |
| Chiba Rosai Hospital | Takashi Saegusa |
| Chiba Tokushukai Hospital | Isao Kitahara |
| Chidoribashi Hospital | Yasushi Ejima |
| Chigasaki Municipal Hospital | Hiroshi Tanaka |
| Chikamori Hospital | Satoru Hayashi |
| Chubu Rousai Hospital | Kazuyoshi Hattori |
| Chugoku Rosai Hospital | Shinji Okita |
| Chutoen General Medical Center | Toshikazu Ichihashi |
| Daiichitowakai Hospital | Tsugumichi Ichioka |
| Daiyukai General Hospital | Shinichi Shirakami |
| Date Red Cross Hospital | Takeshi Matsuoka |
| Dokkyo Medical University Koshigaya Hospital | Akio Hyodo |
| Doutou Neurosurgical Hospital | Teruo Kimura |
| Ebina General Hospital | Tomonori Kobayashi |
| Ehime Prefectural Central Hospital | Kanehisa Kohno |
| Fuchu Hospital | Kazunori Yamanaka |
| Fuji City General Hospital | Akira Morooka |
| Fujii Masao Memorial Hospital | Nozomi Mori |
| Fujii Neurosurgical Hospital | Hideo Kunimine |
| Fujimoto General Hospital | Kazutaka Yatsushiro |
| Fujita General Hospital | Masahiro Satoh |
| Fujita Health University Hospital | Ichiro Nakahara |
| Fujiyoshida Municipal Hospital | Syougo Imae |
| Fukaya Red Cross Hospital | Hirochiyo Wada |
| Fukuchiyama City Hospital | Mamoru Murakami |
| Fukui Katsuyama General Hospital | Masanori Kabuto |
| Fukuoka City Hospital | Katsuyuki Hirakawa |
| Fukuoka Seisyukai Hospital | Isao Inoue |
| Fukuoka Tokushukai Medical Center | Hidenori Yoshida |
| Fukuoka University Chikushi Hospital | Kiyoshi Kazekawa |
| Fukuoka University Hospital | Masani Nonaka |
| Fukuoka Wajiro Hospital | Kouzou Fukuyama |
| Fukuokashinmizumaki Hospiral | Shigenari Kin |
| Fukushima Medical University Hospital | Kiyoshi Saito |
| Fukushima Red Cross Hospital | Yoichi Watanabe |
| Fukuyama City Hospital | Tadashi Arisawa |
| Furukawaseiryou Hospital | Kou Takahashi |
| Gifu Municipal Hospital | Tetsuya Tanigawara |
| Gifu Prefectural Tajimi Hospital | Junki Ito |
| Gifu University Hospital | Toru Iwama |
| Hachisuga Hospital | Kei Hisada |
| Hakodate Central General Hospital | Makoto Takeda |
| Hakodate Municipal Hospital | Jun Niwa |
| Hakodate Neurosurgical Hospital | Mikio Nishiya |
| Hakujyuji Hospital | Shuji Hayashi |
| Hamamatsu City Rehabilitation Hospital | Ichiro Fujishima |
| Hamamatsu Medical Center | Teiji Nakayama |
| Hamamatsu Rosai Hospital | Yoshihiko Watanabe |
| Hamanomachi Hospital | Koichirou Matsukado |
| Hanwa Memorial Hospital | Takamichi Yuguchi |
| Harasanshin Hospital | Tadahisa Shono |
| Hata Kenmin Hospital | Hiroyuki Nishimura |
| Hayashi Hospital | Jyunya Hayashi |
| Hekinan Municipal Hospital |  |
| Higashihiroshima Medical Center | Keisuke Migita |
| Higashiosaka City Medical Center | Kazuhiro Yokoyama |
| Higashisumiyoshi Morimoto Hospital | Naofumi Isono |
| Higashitotsuka Memorial Hospital | Satoshi Utsuki |
| Higashiyamato Hospital | Hirotoshi Ohtaka |
| Himi Municipal Hospital | Takata Hisashi |
| Hirosaki Stroke and Rehabilitation Center | Takamitsu Uchizawa |
| Hirosaki University Hospital | Hiroki Ohkuma |
| Hiroshima City Hiroshima Citizens Hospital | Shigeki Nishino |
| Hiroshima Prefectural Hospital | Atsushi Tominaga |
| Hiroshima Red Cross Hospital & Atomic Bomb Survivors Hospital | Masayuki Sumida |
| Hiroshima University Hospital | Kaoru Kurisu |
| HITO Medical Center | Naoki Shinohara |
| Hokkaido University Hospital | Kiyohiro Houkin |
| Hokushikai Megumino Hospital | Mitsunobu Kaijima |
| Hokuto Hospital | Kazumi Nitta |
| Hospital of the University of Occupational and Environmental Health | Junkoh Yamamoto |
| Hyogo College of Medicine | Shinichi Yoshimura |
| Hyogo Prefectural Awaji Medical Center | Yoshio Sakagami |
| Hyogo Prefectural Kakogawa Medical Center | Hideo Aihara |
| Hyogo Prefectural Nishinomiya Hospital | Takayuki Sakaki |
| Ibaraki Prefectural Central Hospital | Hiroko Oyama |
| Ibaraki Seinan Medical Center Hospital | Keishi Fujita |
| Iida Municipal Hospital | Sumio Kobayashi |
| Imari Arita Kyoritsu Hospital | Nobuaki Momozaki |
| Ina Central Hospital | Atsushi Sato |
| Inagi Municipal Hospital | Hideki Murakami |
| Inazawa Municipal Hospital | Masahito Hara |
| Institute of Brain and Blood Vessels Mihara Memorial Hospital | Akazi Kazunori |
| Ise Red Cross Hospital | Fumitaka Miya |
| Ishikawa Prefectural Central Hospital | Hisato Minamide |
| Ishikiriseiki Hospital | Tsuyoshi Inoue |
| Ishinkai Yao General Hospital | Shinichiro Kurokawa |
| Ishinomaki Red Cross Hospital | Syuichi Ishikawa |
| Itabashi Chuo Medical Center | Naohisa Miura |
| Itami Kousei Neurosurgical Hospital | Shinya Noda |
| Iwaki Kyoritsu General Hospital | Shoji Mashiyama |
| Iwata Municipal General Hospital | Shinji Amano |
| Iwate Medical University Hospital | Kuniaki Ogasawara |
| Iwate Prefectural Central Hospital | Takayuki Sugawara |
| Iwate Prefectural Isawa Hospital | Yukihiko Shimizu |
| Iwate Prefectural Iwai Hospital | Keiichi Saito |
| Iwate Prefectural Kuji Hospital | Kazuyuki Miura |
| Iwate Prefectural Ninohe Hospital | Akinori Yabuta |
| Iwate Prefectural Oofunato Hospital | Tatumi Yamanome |
| Izumi General Medical Center | Hiroshi Seto |
| Izumi Hospital | Makoto Hasebe |
| Izumino Hospital | Hikaru Mizobuchi |
| JA Akita Kouseiren Oomagarikousei Medical center | Junkoh Sasaki |
| JA Toride Medical Center | Shin Tsuruoka |
| Japanese Red Cross Akita Hospital | Keiichi Nishimaki |
| Japanese Red Cross Asahikawa Hospital | Katsumi Takizawa |
| Japanese Red Cross Fukui Hospital | Hiroki Toda |
| Japanese Red Cross Fukuoka Hospital | Hitoshi Tsugu |
| Japanese Red Cross Kitami Hospital | Nozomi Suzuki |
| Japanese Red Cross Kochi Hospital | Takeshi Kohno |
| Japanese Red Cross Kumamoto Hospital | Shu Hasegawa |
| Japanese Red Cross Maebashi Hospital | Ken Asakura |
| Japanese Red Cross Medical Center | Ichiro Suzuki |
| Japanese Red Cross Society Azumino Hospital | Masaki Miyatake |
| Japanese Red Cross Society Hachinohe Hospital | Hiromu Konno |
| Japanese Red Cross Takayama Hospital | Katsunobu Takenaka |
| JCHO Chukyo Hospital | Akira Ikeda |
| JCHO Hitoyoshi Medical Center | Keizou Yamamoto |
| JCHO Isahaya General Hospital | Yoshihiro Nishiura |
| JCHO Kobe Central Hospital | Keigo Matsumoto |
| JCHO Kumamoto General Hospital | Kazunari Koga |
| JCHO Kyushu Hospital | Satoshi Inoha |
| JCHO Nankai Medical Center | Masaki Morisige |
| JCHO Tokuyama Central Hospital | Kunihiko Harada |
| JCHO Tokyo Takanawa Hospital | Hirofumi Hiyama |
| JCHO Tokyo Yamate Medical Center | Yasuaki Takeda |
| JCHO Yokohama Chuo Hospital | Taturou Mori |
| Jinmeikai Akiyama Neurosurgical Hospital | Takekazu Akiyama |
| Juntendo Tokyo Koto Geriatric Medical Center | Osamu Okuda |
| Juntendo University Hospital | Hajime Arai |
| Juntendo University Nerima Hospital |  |
| Juzenkai Hospital |  |
| Kaetsu Hospital | Kazuaki Awamori |
| Kaga Medical Center | Naoki Shirasaki |
| Kagawa Rosai Hospital | Kimihiro Yoshino |
| Kagawa University Hospital | Atsushi Shindo |
| Kagoshima City Hospital | Kazuho Hirahara |
| Kagoshima Prefectural Kanoya Medical Center | Shunichi Tanaka |
| Kagoshima Tokushukai Hospital | Teruaki Kawano |
| Kagoshima University Hospital | Kazunori Arita |
| Kainan Hospital |  |
| Kamagaya General Hospital | Hiroaki Sawaura |
| Kameda Medical Center | Hiromu Hadeishi |
| Kamiiida Daiichi General Hospital | Yoichi Uozumi |
| Kan-Etsu Hospital | Masahiko Tanaka |
| Kanazawa Medical University Hospital | Shunsuke Shiraga |
| Kanazawa Neurosurgical Hospital | Shuji Sato |
| Kanazawa University Hospital | Mitsutoshi Nakada |
| Kaneda Hospital | Kimihisa Kinoshita |
| Kano Hospital | Nakazawa Kazutomo |
| Kansai Electric Power Hospital | Yasuhiro Fujimoto |
| Kansai Medical University Hospital | Kunikazu Yoshimura |
| Kansai Medical University Takii Hospital | Masaaki Iwase |
| Kanto Neurosurgical Hospital | Shinichi Yagi |
| Kanto Rosai Hospital | Atsushi Tsuchiya |
| Kasai Shoikai Hospital | Junichi Harashina |
| Kasaoka Daiichi Hospital | Akira Watanabe |
| Kashiwaba Neurosurgical Hospital | Sadao Kaneko |
| Kasugai Municipal Hospital | Naoto Kuwayama |
| Kawasaki Hospital | Junya Hayashi |
| Kawasaki Medical School Hospital | Masaaki Uno |
| Kazuno Kosei Hospital | Masayuki Sasou |
| Keiju Medical Center | Sotaro Higashi |
| Keio University Hospital | Kazunari Yoshida |
| Kenwakai Hospital | Masakazu Kitahara |
| Kenwakai Otemati Hospital | Kenwakai Otemati Hospital |
| Kieikai Hospital | Satoshi Suzuki |
| Kimitsu Chuo Hospital | Sumio Suda |
| Kindai University Hospital | Amami Kato |
| Kindai University Sakai Hospital | Yusaku Nakamura |
| Kiryu Kosei General Hospital | Satoshi Magarisawa |
| Kishiwada City Hospital | Kenji Hashimoto |
| Kishiwada Tokushukai Hospital | Hiroyuki Matsumoto |
| Kita-Harima Medical Center | Hirotoshi Hamaguchi |
| Kitakami Saiseikai Hospital | Tomohiko Satou |
| Kitakyushu General Hospital | Masaru Idei |
| Kitakyushu Municipal Medical Center | Haruhisa Tsukamoto |
| Kitamurayama Hospital | Eiichiro Kamatsuka |
| Kitasato University Hospital | Toshihiro Kumabe |
| Kobari General Hospital | Naoaki Sato |
| Kobayashi Neurosurgical Neurological Hospital | Yasuyuki Toba |
| Kobe City Medical Center General Hospital | Nobuyuki Sakai |
| Kobe Ekisaikai Hospital | Takashi Tominaga |
| Kobe Red Cross Hospital | Haruo Yamashita |
| Kobe University Hospital | Eiji Kohmura |
| Kochi Health Sciences Center | Masanori Morimoto |
| Kochi Medical School Hospital | Tetsuya Ueba |
| Kofu Neurosurgical Hospital | Toyoaki Shinohara |
| Kohka Public Hospital | Kazuyoshi Watanabe |
| Kohnan Hospital | Hidenori Endo |
| Kohsei General Hospital | Kenjirou Hujiwara |
| Kokura Memorial Hospital | Ichiro Nakahara |
| Komaki City Hospital | Toshinori Hasegawa |
| Komatsu Municipal Hospital | Hisashi Nitta |
| Komoro Kosei General Hospital | Kuroyanagi Takayuki |
| Konan Kosei Hospital | Nobuhiko Mizutani |
| Koshigaya Municipal Hospital | Akira Tsunoda |
| Koto Memorial Hospital | Fumio Suzuki |
| Kouseikai Takai Hospital | Tetsuya Morimoto |
| Koyama Memorial Hospital | Takuya Kawai |
| Kugayama Hospital | Mitsuyuki Fujitsuka |
| Kumamoto City Hospital | Akira Takada |
| Kumamoto Rousai Hospital | Hiromasa Tsuiki |
| Kumamoto University Hospital | Junichi Kuratsu |
| Kurashiki Central Hospital | Masaki Chin |
| Kurashiki Heisei Hospital | Hidemichi Sasayama |
| Kurosawa Hospital | Shigehiro Ohmori |
| Kurosishi General Hospital | Seiko Hasegawa |
| Kurosu Hospital | Kazuhiro Kikuchi |
| Kurume University Hospital | Motohiro Morioka |
| Kushiro Rosai Hospital | Masanori Isobe |
| Kyorin University Hospital | Hiroki Yoshida |
| Kyoritsu Hospital | Masayuki Yokota |
| Kyoto City Hospital | Nozomu Murai |
| Kyoto Katsura Hospital | Yasumasa Yamamoto |
| Kyoto Min-Iren Chuo Hospital | Nobuhito Mori |
| Kyoto Okamoto Memorial Hospital | Minoru Kidooka |
| Kyoto Second Red Cross Hospital | Hiroshi Tenjin |
| Kyoto University Hospital | Susumu Miyamoto |
| Kyoto Yamashiro General Medical Center | Yoshihiro Iwamoto |
| Kyushu Central Hospital of the Mutual Aid Association of Public School Teachers | Hitonori Takaba |
| Kyushu Rosai Hospital | Sei Haga |
| Kyushu University Hospital | Koji Iihara |
| Makita General Hospital | Yoshinori Arai |
| Maruko Central Hospital | Toshiyuki Tsukada |
| Matsudo City Hospital | Hirohide Karasudani |
| Matsushita Memorial Hospital | Kenji Hashimoto |
| Matsuyama Shimin Hospital | Masakazu Suga |
| Mazda Hospital | Kawamoto Yukihiko |
| Meisei Hospital | Hiroaki Fujiwara |
| Meiseikai Abashiri Neurosurgical Rehabilitation Hospital | Naoto Izumi |
| Meitetsu Hospital | Youtarou Takeuchi |
| Midorigaoka Hospital | Motohiro Arai |
| Mie University Hospital | Hidenori Suzuki |
| Mimihara General Hospital | Shinji Okumura |
| Minamata City General Hospital and Medical Center | Hiromasa Tsuiki |
| Minamisoma City General Hospital | Tomoyoshi Oikawa |
| Minato Medical Coop-Kyoritsu General Hospital | Hisashi Tanaka |
| Mito Kyodo General Hospital | Yasushi Shibata |
| Mitoyo General Hospital | Tetsuya Masaoka |
| Mitsugi General Hospital | Takashi Matsuoka |
| Mitsuwadai General Hospital | Masahiko Kasai |
| Miyake Neurosurgical Hospital | Hitoshi Miyake |
| Miyakonojo Medical Association Hospital | Hajime Ohta |
| Miyoshi Central Hospital | Osamu Hamasaki |
| Moriguchi-Ikuno Memorial Hospital | Misao Nishikawa |
| Morioka Red Cross Hospital | Naohiko Kubo |
| Munakata Suikokai General Hospital | Yosimasa Kinosita |
| Muroran City General Hospital | Hiroshi Ooyama |
| Musashino General Hospital | Hiroyuki Kaidu |
| Nagahama City Hospital | Tarou Komuro |
| Nagano Children's Hospital | Hiroaki Shigeta |
| Nagano Municipal Hospital | Yoshikazu Kusano |
| Nagaoka Chuo General Hospital | Shigekazu Takeuchi |
| Nagasaki University Hospital | Takayuki Matsuo |
| Nagasakiken Shimabara Hospital | Yoshiharu Tokunaga |
| Nagoya Central Hospital | Norimoto Nakahara |
| Nagoya City East Medical Center | Nobukazu Hashimoto |
| Nagoya City University Hospital | Mitsuhito Mase |
| Nagoya Daini Red Cross Hospital | Keizo Yasui |
| Nagoya Memorial Hospital | Junpei Yoshimoto |
| Nagoya University Hospital | Toshihiko Wakabayashi |
| Naha City Hospital | Jin Momoji |
| Nakamura Memorial Hospital | Kenji Kamiyama |
| Nakamura Memorial South Hospital | Koji Oka |
| Nakatsu Municipal Hospital | Hiromichi Koga |
| Nakatsu Neurosurgical Hospital | Kazuya Morimoto |
| Nanbu Tokushukai Hospital | Tsutomu Kadekaru |
| Nara Medical University Hospital | Hiroyuki Nakase |
| Nara Prefectural Nara Hospital | Junichi Iida |
| Narita Red Cross Hospital | Michio Nakamura |
| National Cerebral and Cardiovascular Center | Hiroharu Kataoka |
| Nayoro City Hospital | Naoki Tokumitsu |
| NHO Beppu Medical Center | Yasuyuki Nagai |
| NHO Chiba Medical Center | Hirokazu Tanno |
| NHO Disaster Medical Center | Hiroyuki Masaoka |
| NHO Fukuoka Higashi Medical Center | Hiroshi Nakane |
| NHO Hamada Medical Center | Takato Kagawa |
| NHO Himeji Medical Center | Masaaki Saiki |
| NHO Hokkaido Medical Center | Satoshi Ushikoshi |
| NHO Iwakuni Clinical Center | Kotaro Ogihara |
| NHO Kagoshima Medical Center | Junichi Imamura |
| NHO Kanmon Medical Center | Katsuhiro Yamashita |
| NHO Kyushu Medical Center | Akira Nakamizo |
| NHO Minami Wakayama Medical Center | Yoshinari Nakamura |
| NHO Nagasaki Kawatana Medical Center | Ei-Ichirou Urasaki |
| NHO Nagoya Medical Center | Noriyuki Suzaki |
| NHO Nara Medical Center | Hidehiro Hirabayashi |
| NHO Niigata Hospital | Chiaki Takahashi |
| NHO Okayama Medical Center | Youichirou Namba |
| NHO Osaka National Hospital | Kazuo Hashikawa |
| NHO Osakaminami Medical Center | Tomonori Yamada |
| NHO Sendai Medical Center | Masayuki Ezura |
| NHO Shikoku Medical Center for Children and Adults | Kazuyuki Kuwayama |
| NHO Shinshu Ueda Medical Center | Keiichi Sakai |
| NHO Shizuoka Medical Center | Katsuhiro Kuroda |
| NHO Takasaki General Medical Center | Hideyuki Kurihara |
| NHO Tochigi Medical Center | Masayuki Ishihara |
| NHO Toyohashi Medical Center | Hideki Sakai |
| NHO Ureshino Medical Center | Masayuki Miyazono |
| NHO Yokohama Medical Center | Kosuke Miyahara |
| Nihon University Itabashi Hospital | Atsuo Yoshino |
| Niigata Cancer Center Hospital | Hideaki Takahashi |
| Niigata City General Hospital | Akihiko Saito |
| Niigata Neurosurgical Hospital | Hiroyuki Arai |
| Niigata Prefectural Central Hospital | Igarashi Michitoku |
| Niigata Tokamachi Hospital | Mitsuo Kouno |
| Niigata University Medical & Dental Hospital | Osamu Onodera |
| Nippon Medical School Chiba Hokusoh Hospital | Shiro Kobayashi |
| Nipponbashi Hospital | Shunichi Yoneda |
| Nishiagatsuma Welfare Hospital | Hiroshi Kusunoki |
| Nishikobe Medical Center | Naoya Takeda |
| Nishinomiya Kyoritsu Neurosurgical Hospital | Hiroji Miyake |
| Nishio Municipal Hospital | Toshio Yokoe |
| Nishitokyo Central General Hospital | Tatsuya Nakamura |
| Nishiwaki Municipal Hospital |  |
| Nissan Tamagawa Hospital | Takayuki Kubodera |
| Nitobe Memorial Nakano General Hospital | Mitsuhiko Hokari |
| Noshiro Kosei Medical Center | Yasunari Otawara |
| Noto General Hospital | Cheho Park |
| Nozaki Tokushukai Hospital | Hidemitu Nakagawa |
| NTT Medical Center Tokyo | Kensuke Kawai |
| Obara Hospital | Souichi Obara |
| Obase Hospital | Haruki Takahashi |
| Obihiro Kosei General Hospital | Masafumi Ohtaki |
| Odate Municipal General Hospital | Atsuya Okubo |
| Ogaki Tokushukai Hospital | Katsuhiko Hayashi |
| Ohnishi Neurological Center | Hideyuki Ohnishi |
| Ohta Nishinouchi Hospital | Masahisa Kawakami |
| Oita Prefectural Hospital | Yu Takeda |
| Oitaken Koseiren Tsurumi Hospital | Akihiko Kaga |
| Oitaoka Hospital | Yutaka Yamaguchi |
| Okaya City Hospital | Ryoichi Hayashi |
| Okayama City Hospital | Koji Tokunaga |
| Okayama Kyokuto Hospital | Hiroyuki Nakashima |
| Okayama Kyoritsu General Hospital |  |
| Okayama Saiseikai General Hospital | Yasuyuki Miyoshi |
| Okayama University Hospital | Isao Date |
| Okinawa Kyodo Hospital | Atusi Kimoto |
| Okinawa Miyako Hospital | Satoshi Yamamoto |
| Okinawa Prefectural Hokubu Hospital | Toshimitsu Uchihara |
| Okinawa Prefectural Nanbu Medical Center / Nanbu Child Medical Center | Tomoaki Nagamine |
| Okinawa Red Cross Hospital | Masahiro Noha |
| Okinawatokushuukai Uwajimatokushukai Hospital | Hiromichi Sadashima |
| Okitama Public General Hospital | Toshihiko Kinjo |
| Ome Municipal General Hospital | Osamu Tao |
| Omihachiman Community Medical Center | Masayuki Nakajima |
| Omori Red Cross Hospital | Akira Isoshima |
| Omuta City Hospital | Kouichi Kuramoto |
| Onomichi Municipal Hospital | Shigeru Daido |
| Osaka City General Hospital | Yoshiyasu Iwai |
| Osaka City University Hospital | Kenji Ohata |
| Osaka Kouseinenkin Hospital |  |
| Osaka Medical Center for Cancer and Cardiovascular Diseases | Manabu Kinoshita |
| Osaka Medical College Hospital | Toshihiko Kuroiwa |
| Osaka Neurological Institute | Akatsuki Wakayama |
| Osaka Neurosurgical Hospital | Naoki Hayashi |
| Osaka Red Cross Hospital | Kohsuke Yamashita |
| Osaka Saiseikai Ibaraki Hospital | Yasunobu Gotou |
| Osaka University Hospital | Kouich Iwatsuki |
| Osaki Citizen Hospital | Yoshida Masahiro |
| Ota Memorial Hospital |  |
| Otaru Chuo Hospital | Nobuaki Kobayasi |
| Otaru General Hospital | Yoshimasa Niiya |
| Otaru Municipal Medical Center for Brain Cardiovascular and Mental disorders | Syouji Mabuchi |
| Otsu City Hospital | Motohiro Takayama |
| Otsu Red Cross Hospital | Masaaki Saiki |
| Rakuwakai Otowa Hospital | Kazuo Yamamoto |
| Research Institute for Brain and Blood Vessels-Akita | Junta Moroi |
| Rinku General Medical Center |  |
| Rumoi Central Clinic | Susumu Suzuki |
| Saga University Hospital | Tatsuya Abe |
| Saga-Ken Medical Centre Koseikan | Hiroshi Sugimori |
| Sagamihara Kyodo Hospital | Masato Sugitani |
| Saiseikai Fukuoka General Hospital | Akio Ookura |
| Saiseikai Futsukaichi Hospital | Naoko Fujimura |
| Saiseikai Gose Hospital |  |
| Saiseikai Imabari Hospital | Osamu Nishizaki |
| Saiseikai Kawaguchi General Hospital | Sumio Isimaru |
| Saiseikai Kumamoto Hospital | Toru Nishi |
| Saiseikai Kurihashi Hospital | Hiroshi Wanihuchi |
| Saiseikai Kyoto Hospital | Nobukuni Murakami |
| Saiseikai Matsusaka General Hospital | Hiroto Murata |
| Saiseikai Nagasaki Hospital | Naoki Kitagawa |
| Saiseikai Noe Hospital | Katsuhiko Kono |
| Saiseikai Toyama Hospital | Michiya Kubo |
| Saiseikai Ustunomiya Hospital | Masashi Nakatsukasa |
| Saiseikai Yahata General Hospital | Yuji Okamoto |
| Saiseikai Yokohamashi Tobu Hospital | Makoto Inaba |
| Saitama Cardiovascular and Respiratory Center | Hidetoshi Ooigawa |
| Saitama City Hospital | Atsuhiro Kojima |
| Saitama Medical Center | Kyoichi Nomura |
| Saitama Medical University Hospital | Takamitsu Fujimaki |
| Saitama Red Cross Hospital | Kenji Yamamoto |
| Saito Memorial Hospital | Osamu Fukuda |
| Sakai City Medical Center | Yoshikazu Nakajima |
| Saku Central Hospital | Kazuyuki Kouno |
| Saku Central Hospital Advanced Care Center | Takaaki Yoshida |
| Sanseikai Kanemaru Neurosurgery Hospitai | Reizou Kanemaru |
| Sanyudo Hospital | Yohei Kudoh |
| Sapporo Azabu Neurosurgical Hospital | Toshitaka Nakamura |
| Sapporo City General Hospital | Masayoshi Takigami |
| Sapporo Higashi Tokushukai Hospital | Shogo Nishi |
| Sapporo Medical University Hospital | Nobuhiro Mikuni |
| Sapporo Shiroishi Memorial Hospital | Akira Takahashi |
| Sapporoteishinkai Hospital | Rokuya Tanikawa |
| Sasebo Chuo Hospital | Seisaburo Sakamoto |
| Sasebo City General Hospital | Makio Kaminogo |
| Secomedic Hospital | Seiichiro Hoshi |
| Seikeikai Hospital | Yoshinari Okumura |
| Seirei Memorial Hospital | Shinichi Okabe |
| Seirei Mikatahara General Hospital | Haruhiko Sato |
| Seiwakai Wada Hospital | Shiro Miyata |
| Sekishinkai Kawasakisaiwai Hospital | Kotaro Tsumura |
| Sendai City Hospital | Hiroshi Karibe |
| Sendai East Neurosurgical Hospital | Noriaki Watabe |
| Shiga University of Medical Science Hospital | Kazuhiko Nozaki |
| Shimane Prefectural Central Hospital | Fusao Ikawa |
| Shimizu Hospital | Takashi Yoshida |
| Shimonoseki City Hospital | Ryuji Nakamura |
| Shimotsuga General Hospital | Norifumi Shimoeda |
| Shin Koga Hospital | Tsutomu Hitotsumatsu |
| Shin-Oyama City Hospital | Tomoaki Kameda |
| Shingu Municipal Medical Center | Hiroshi Ishiguchi |
| Shinoda General Hospital | Atsuo Shinoda |
| Shinonoi General Hospital | Masanobu Hokama |
| Shinsapporo Neurosurgical Hospital | Akinori Yamamura |
| Shinseikai Toyama Hospital |  |
| Shinshu University Hospital | Kazuhiro Hongo |
| Shinsuma General Hospital | Takeshi Kondoh |
| Shintakeo Hospital | Makoto Ichinose |
| Shinwakai Yachiyo Hospital | Takashi Inoue |
| Shiroyama Hospital | Kenichi Murao |
| Shizuoka Children's Hospital | Takafumi Wataya |
| Shizuoka City Shizuoka Hospital | Seiji Fukazawa |
| Shonan Kamakura General Hospital | Takahisa Mori |
| Showa Inan General Hospital | Shinsuke Muraoka |
| Showa University Fujigaoka Hospital | Tomoaki Terada |
| Shuto General Hospital | Hirosuke Fujisawa |
| Shuuwa General Hospital | Tsuneo Shishido |
| Souseikai Shin Yoshizuka Hospital | Mayumi Mori |
| South Miyagi Medical Center | Arai Hiroaki |
| Southern Tohoku General Hospital | Shinjitsu Nishimura |
| Southern Tohoku Hospital | Zenichiro Watanabe |
| St. Marianna University School of Medicine | Yuichiro Tanaka |
| St. Lukes International Hospital | Yasunari Niimi |
| St. Mary’s Hospital | Susumu Nakashima |
| Steel Memorial Yawata Hospital | Akira Nakamizo |
| Suiseikai Kajikawa Hospital | Shinichi Wakabayashi |
| Suisyoukai Murata Hospital | Kazuhito Nakamura |
| Suwa Central Hospital | Hiroki Sato |
| Suwa Red Cross Hospital | Yukinari Kakizawa |
| Tachikawa General Hospital | Hiroki Takano |
| Takamatsu Municipal Hospital | Norihito Shirakawa |
| Takamatsu Red Cross Hospital | Masahiro Kagawa |
| Takarazuka City Hospital | Eiichiro Mabuchi |
| Takarazuka Daiichi Hospital | No |
| Takatsuki General Hospital | Kazusige Maeno |
| Takeda General Hospital | Takayuki Koizumi |
| Takeda Hospital | Warou Taki |
| Takikawa Neurosurgical Hospital | Yusuke Nakagaki |
| Tane General Hospital | Kazuyuki Tane |
| Tannan regional medical center |  |
| Tanushimaru Central Hospital | Hiromichi Ooishi |
| Teinekeijinkai Hospital | Katsuyuki Asaoka |
| Tenri Hospital | Yoshinori Akiyama |
| Tenshindo Hetsugi Hospital | Tadao Kawamura |
| Teraoka Memorial Hospital | Atumi Takenobu |
| The Veritas Hospital | Masayuki Yokota |
| Tobata Kyoritsu Hospital | Takehisa Tuji |
| Tohoku University Hospital | Teiji Tominaga |
| Tokai University Hachioji Hospital | Masami Shimoda |
| Tokai University Hospital | Mitsunori Matsumae |
| Toki General Hospital | Shinji Noda |
| Tokuda Neurosurgical Hospital | Koiti Moroki |
| Tokushima Prefectural Kaifu Hospital | Hirofumi Oka |
| Tokushima Prefecture Naruto Hospital | Masahito Agawa |
| Tokushima Red Cross Hospital | Hajimu Miyake |
| Tokushima University Hospital | Junichiro Satomi |
| Tokyo Dental College Ichikawa General Hospital | Masateru Katayama |
| Tokyo General Hospital | Shinichi Numazawa |
| Tokyo Medical and Dental University Hospital | Taketoshi Maehara |
| Tokyo Medical University Hachioji Medical Center | Hiroyuki Jimbo |
| Tokyo Medical University Hospital | Michihiro Kohno |
| Tokyo Metropolitan Children's Medical Center | Satoshi Ihara |
| Tokyo Metropolitan Geriatric Hospital and Institute of Gerontology | Koji Matuoka |
| Tokyo Metropolitan Health and Medical Treatment Corporation Ohkubo Hospital | Oikawa Akihiro |
| Tokyo Metropolitan Hiroo Hospital | Kensaku Yoshida |
| Tokyo Metropolitan Tama Medical Center | Takahiro Oota |
| Tokyo Saiseikai Central Hospital | Haruhiko Hoshino |
| Tokyo Teisin Hospital | Makoto Noguchi |
| Tokyo Women's Medical University Hosptal | Takakazu Kawamata |
| Tomakomaihigashi Hospital | Youichi Hashimoto |
| Tomei Atsugi Hospital | Keiichirou Onitsuka |
| Tominaga Hospital | Masahiko Kitano |
| Tomishiro Central Hospital | Jae-Hyun Son |
| Tonami General Hospital | Toru Masuoka |
| Tosei General Hospital | Naoki Koketsu |
| Tottori Municipal Hospital | Keiichi Akatsuka |
| Tottori University Hospital | Masamichi Kurosaki |
| Toyama City Hospital | Miyamori Tadao |
| Toyama Prefectural Central Hospital | Hiroaki Hondo |
| Toyama Red Cross Hospital | Kazumasa Yamatani |
| Toyama University Hospital | Satoshi Kuroda |
| Toyohashi Municipal Hospital | Hirofumi Oyama |
| Toyokawa City Hospital | Takashi Matsumoto |
| Toyooka Hospital | Junji Koyama |
| Toyota Kosei Hospital | Ogura Koichiro |
| Tsuchiura Kyodo General Hospital | Shinji Yamamoto |
| Tsuchiura Kyodo Hospital Namegata District Medical Center | Hitoshi Tabata |
| Tsukazaki Hospital |  |
| Tsukuba Medical Center Hospital | Kazuya Uemura |
| Tsuruoka Municipal Shonai Hospital | Kazuhiko Sato |
| Tsuyama Chuo Hospital | Hideyuki Yoshida |
| Ube-Kohsan Central Hospital | Takafumi Nishizaki |
| Uki General Hospital | Hiroshi Egami |
| University of Fukui Hospital | Osamu Yamamura |
| University of Miyazaki Hospital | Hideo Takeshima |
| University of the Ryukyus Hospital | Shogo Ishiuchi |
| University of Tsukuba Hospital | Akira Matsumura |
| University of Yamanashi Hospital | Hiroyuki Kinouchi |
| Urasoe General Hospital | Susumu Mekaru |
| Ushiku Aiwa General Hospital | Mikihiko Takeshita |
| Ushioda General Hospital | Hitoshi Ozawa |
| Usuikai Tano Hospital |  |
| Uwajima City Hospital | Kiichiro Zenke |
| Wakakusa Daiichi Hospital | Takeshi Matsuyama |
| Wakayama Medical University Hospital | Naoyuki Nakao |
| Wakayama Rosai Hospital | Toshikazu Kuwata |
| Wakayama-Seikyo Hospital | Teruyuki Habu |
| Wakkanai Teishinkai Hospital | Tomoyoshi Okumura |
| Yaizu City Hospital | Seiya Takehara |
| Yamagata City Hospital Saiseikan | Rei Kondo |
| Yamagata Prefectural Central Hospital | Takashi Kumagai |
| Yamagata Prefectural Shinjo Hospital | Keiten So |
| Yamagata Saisei Hospital | Sunao Takemura |
| Yamagata University Hospital | Sonoda Yukihiko |
| Yamaguchi Prefectural Grand Medical Center | Manabu Urakawa |
| Yamaguchi Red Cross Hospital | Yasuhiro Hamada |
| Yamaguchi University Hospital | Michiyasu Suzuki |
| Yamanashi Kosei Hospital | Mikito Uchida |
| Yamanashi Prefectural Central Hospital | Hidehito Koizumi |
| Yamanashi Red Cross Hospital | Hiroshi Ozawa |
| Yamato Municipal Hospital | Masaru Yamada |
| Yao Tokushukai General Hospital | Takashi Tsuruno |
| Yasugi Municipal Hospital | Gen Ishida |
| Yatsuo General Hospital | Ryouichi Masuda |
| Yawata Medical Center | Makoto Kimura |
| Yayoigaoka Kage Hospital | Shinichirou Ishihara |
| Yodogawa Christian Hospital | Masashi Morikawa |
| Yokkaichi Municipal Hospital |  |
| Yokohama Asahi Chuo General Hospital | Sachio Suzuki |
| Yokohama City Minato Red Cross Hospital | Hiroaki Tanaka |
| Yokohama City University Hospital | Hidetoshi Murata |
| Yokohama City University Medical Center | Katsumi Sakata |
| Yokohama Rosai Hospital |  |
| Yokohama Sakae Kyosai Hospital | Motohiro Nomura |
| Yokohamashintoshi Neurosurgecal Hospital | Akihiro Nemoto |
| Yokohamasinmidori Hospital | Sumio Endou |
| Yokosuka City Uwamachi Hospital | Nobuo Hirota |
| Yonezawa City Hospital | Kennji Itou |
| Yoshida Hospital | Hiroaki Minami |
| Yuaikai Hospital | Yoshihumi Teramoto |
